# Structural Brain Network: What is the Effect of LiFE Optimization of Whole Brain Tractography?

**DOI:** 10.3389/fncom.2016.00012

**Published:** 2016-02-16

**Authors:** Shouliang Qi, Stephan Meesters, Klaas Nicolay, Bart M. ter Haar Romeny, Pauly Ossenblok

**Affiliations:** ^1^Sino-Dutch Biomedical and Information Engineering School, Northeastern UniversityShenyang, China; ^2^Academic Center for Epileptology Kempenhaeghe and Maastricht UMC+Heeze, Netherlands; ^3^Department of Biomedical Engineering, Eindhoven University of TechnologyEindhoven, Netherlands; ^4^Department of Mathematics and Computer Science, Eindhoven University of TechnologyEindhoven, Netherlands

**Keywords:** structural connectivity, diffusion-weighted MRI, tractography, brain network, connectome

## Abstract

Structural brain networks constructed based on diffusion-weighted MRI (dMRI) have provided a systems perspective to explore the organization of the human brain. Some redundant and nonexistent fibers, however, are inevitably generated in whole brain tractography. We propose to add one critical step while constructing the networks to remove these fibers using the linear fascicle evaluation (LiFE) method, and study the differences between the networks with and without LiFE optimization. For a cohort of nine healthy adults and for 9 out of the 35 subjects from Human Connectome Project, the *T*_1_-weighted images and dMRI data are analyzed. Each brain is parcellated into 90 regions-of-interest, whilst a probabilistic tractography algorithm is applied to generate the original connectome. The elimination of redundant and nonexistent fibers from the original connectome by LiFE creates the optimized connectome, and the random selection of the same number of fibers as the optimized connectome creates the non-optimized connectome. The combination of parcellations and these connectomes leads to the optimized and non-optimized networks, respectively. The optimized networks are constructed with six weighting schemes, and the correlations of different weighting methods are analyzed. The fiber length distributions of the non-optimized and optimized connectomes are compared. The optimized and non-optimized networks are compared with regard to edges, nodes and networks, within a sparsity range of 0.75–0.95. It has been found that relatively more short fibers exist in the optimized connectome. About 24.0% edges of the optimized network are significantly different from those in the non-optimized network at a sparsity of 0.75. About 13.2% of edges are classified as false positives or the possible missing edges. The strength and betweenness centrality of some nodes are significantly different for the non-optimized and optimized networks, but not the node efficiency. The normalized clustering coefficient, the normalized characteristic path length and the small-worldness are higher in the optimized network weighted by the fiber number than in the non-optimized network. These observed differences suggest that LiFE optimization can be a crucial step for the construction of more reasonable and more accurate structural brain networks.

## Introduction

At the macroscale, the structural brain network comprises anatomically distinct brain regions mathematically defined as nodes (vertices) and the structural pathways connecting pairs of regions defined as links (edges; Bullmore and Sporns, 2009; Craddock et al., 2013). The concept of structural brain networks (Sporns et al., 2005) has gained a lot of popularity recently, because it provides us with an anatomical and physiological substrate of brain functions and helps to understand how the brain structures shape functional interactions (Sporns, 2010; Jiang, 2013; Park and Friston, 2013). A variety of organizational and topological properties of structural brain networks has been found, such as hubs, the rich-club organization, hierarchical modularity, and small-worldness (Sporns et al., 2007; Meunier et al., 2010; Rubinov and Sporns, 2010; van den Heuvel and Sporns, 2011).

Realizing that the brain is a complex network, it is logical to study neurological disorders from a network perspective (Pessoa, 2014). Stam (2014) has pointed out that the failure of hubs might be a common pathway in neurological disorders. Evidences to support this assumption are the lesions which are significantly concentrated in and around hub regions in Alzheimer's disease and schizophrenia (Crossley et al., 2014). Neurological disorders are also considered to be a consequence of the disrupted or dysfunctional brain network architecture (Menon, 2011; Fornito and Bullmore, 2015). Related studies have been generating more sensitive and accurate biomarkers for various brain diseases and driving connectomics to be a paradigm to study neurological disorders (Griffa et al., 2013; Fornito and Bullmore, 2015).

MRI, as a non-invasive and *in vivo* imaging approach, has played an indispensable role in inferring structural brain networks at the macroscale through providing both high resolution 3D T_1_-weighted images and diffusion-weighted MRI (dMRI) data with multiple diffusion gradient schemes (Jones et al., 2013; Bastiani and Roebroeck, 2015). Based on these data, the structural brain network can be constructed through a pipeline mainly including three steps. Firstly, the brain is parcellated into some regions-of-interest, determined as the nodes in the network, by different templates and atlases (Tzourio-Mazoyer et al., 2002; Desikan et al., 2006). Secondly, the structural connections (or edges) are estimated according to the dMRI data. This step comprises two sub-processes: (1) to estimate the fiber orientation density function (fODF) at each voxel through diffusion tensor imaging (DTI) or complex fODF models (Basser et al., 1994; Johansen-Berg and Behrens, 2009); (2) to trace putative white-matter paths by deterministic or probabilistic tractography algorithms (Jbabdi and Johansen-Berg, 2011). The final output of this step is the whole brain tractography, portrayals of white-matter tracts. Thirdly, the adjacency matrix is obtained through counting the number of fibers connecting each pair of nodes (Hagmann et al., 2008; Gong et al., 2009). For an overview of more methodological options, one can refer to the review by Qi et al. (2015).

Whole brain tractography results are usually used to construct the structural network directly, despite the occurrence of redundant and nonexistent fibers (or false positives; Campbell et al., 2005; Bastiani et al., 2012). Especially for the probabilistic algorithms with liberal termination criteria, high sensitivities are gained with the cost of generating more false positives (Dyrby et al., 2007; Qi et al., 2015). Recently, the method of linear fascicle evaluation (LiFE) was proposed by Pestilli et al. (2014) to identify the redundant and nonexistent fibers and remove them from the connectome. LiFE operates like an inverse process of tractography, it uses the fibers generated by whole brain tractography as the inputs to predict the measured diffusion MRI signals in all underlying white matter voxels. A weight is estimated for each fiber, indicating this fiber's contribution to the diffusion data prediction. Only the fibers with positive weights are retained to create an optimized connectome. Pestilli et al. (2014) showed an 80% removal of fibers that were suspected redundant and nonexistent fibers. Eliminating these huge amounts of fibers raises the question whether optimization by LiFE will alter the topological properties of the structural brain network. To our knowledge, the effect of LiFE optimization on the structural brain network has not been investigated before.

The main objective of this study is to assess systematically the effect of LiFE optimization on the structural brain network. To achieve this goal, we firstly remove redundant and nonexistent fibers from the whole brain tractography using LiFE optimization and construct the optimized network. Secondly, we assess the resultant optimized networks in several distinct weighting methods and clarify their relationships. Thirdly, we thoroughly compare the non-optimized network (without LiFE optimization) and the optimized network with regard to the edge weights, the nodal network measures, and the global network measures to reveal their differences.

## Materials and methods

### Data acquisition

The present study included a cohort of nine healthy young adults (3 female, 25 ± 4 aged) without any history of neurological or psychiatric disorders. The study had the approval of the Medical Ethics Committee of Kempenhaeghe (KH 10/02) and all participants gave written informed consent in accordance with the Declaration of Helsinki (2000). Written informed consent was collected from each participant before scanning. All MRI data were collected on a 3.0 T MR scanner (Achieva, Philips Medical Systems, Best). T_1_-weighted images were obtained using a Turbo Field Echo (TFE) sequence (the reconstructed voxel size 1 × 1 × 1 mm, multi slice acquisition of 170 slices, TR 8.4 ms, TE 3.9 ms, flip angle 90°). The dMRI acquisition was performed using the Single-Shot Spin-Echo Echo-Planar Imaging (SE-EPI) sequence in 32 directions with a *b*-value of 1000 s/mm^2^ [TE/TR = 73/6718 ms, SENSE = 2, 112 × 112 matrix, 2.0 mm isotropic resolution, a slice thickness of 2.0 mm, the number of slices = 60, the number of signal averages (NSA) = 2]. Additionally, one non-diffusion weighted (*b* = 0) image volume was also scanned at the beginning of each dataset.

Moreover, data of 9 out of the 35 subjects from MGH Adult Diffusion Data [Human Connectome Project (HCP), http://www.humanconnectome.org/] has been analyzed. One can refer to the HCP website for detailed information on subjects and scanning protocols. For each subject, the diffusion data of 64 directions with a *b*-value of 1000 s/mm^2^ and one non-diffusion weighted (*b* = 0) image volume are extracted and used with T_1_-weighted images together.

### Network construction

**Figure 1:**
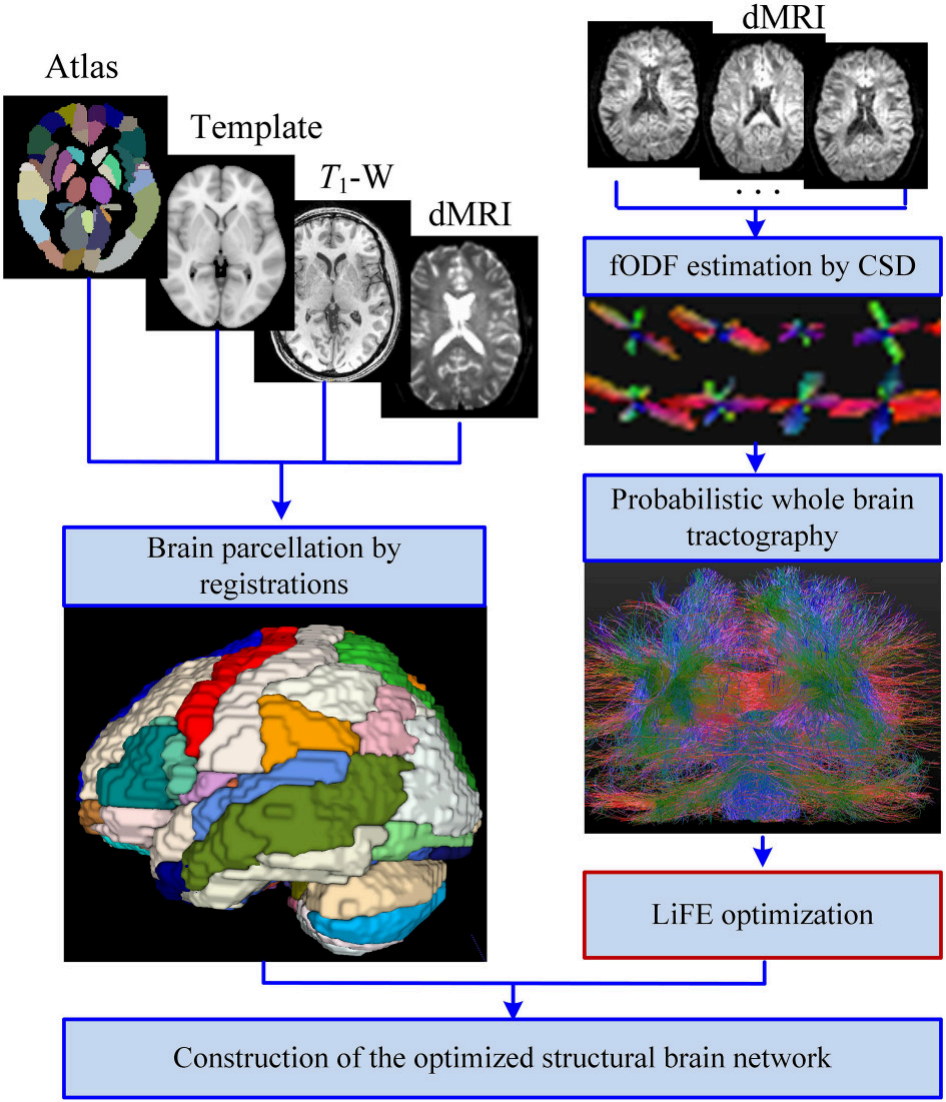
**Improved pipeline for the construction of the structural brain network**. With the inputs of T1-W images, diffusion MRI (dMRI) data, and the automated anatomical labeling (AAL) template and atlas, the brain is parcellated into 90 cortical, and subcortial regions through registrations. Meanwhile, the fiber orientation density function (fODF) of each voxel is estimated by the method of constrained spherical deconvolution (CSD), and a probabilistic whole brain tractography is used to generate the original connectome. Removing the redundant and nonexistent fibers from the original connectome by the linear fascicle evaluation (LiFE) method gives rise to the optimized connectome. Finally the optimized structural brain network is obtained as an adjacency matrix through combining the parcellations and the optimized connectome.

The improved pipeline for construction of structural brain network is shown in Figure 1. In general, the process can be divided into four main steps: (1) Brain parcellation; (2) Whole brain tractography; (3) LiFE optimization; (4) Adjacency matrix construction.

#### Brain parcellation

The automated anatomical labeling (AAL) template and atlas (Tzourio-Mazoyer et al., 2002) are used to parcellate the brain into 90 cortical and subcortical regions. The indices, names and abbreviations of the brain regions are listed in Supplementary Table 1. Each parcellated region is used as a node in the network.

Brain parcellation can be realized through several steps of registration. Firstly, the *T*_1_-weighted image is normalized with the ICBM-152 MNI *T*_1_-template as reference by linear registration of 6° of freedom. Secondly, the *b* = 0 volume is linearly registered to the resultant normalized *T*_1_-weighted images. Thirdly, each set of diffusion data of 32 directions is registered to the resultant *b* = 0 volume, and the output of the transform matrix is applied to rotate the *b* vector (Leemans and Jones, 2009). Fourthly, the ICBM-152 MNI *T*_1_-template is non-linearly registered to normalized *T*_1_-weighted images. Finally, the transformation parameters derived from the last step were inverted and used to warp the AAL atlas to the subject diffusion space. All these registrations are realized by FSL (Jenkinson et al., 2012).

#### Whole brain tractography

The fODF of each voxel is estimated by the method of constrained spherical deconvolution (CSD) with a maximum harmonic order of 6 (Tournier et al., 2007; Descoteaux et al., 2009). Based on these results, a probabilistic tracking algorithm is utilized to realize the whole brain tractography using MRtrix (Tournier et al., 2012). We set the step size at 0.2 mm, the white matter volume as the seed mask, the fODF amplitude cutoff at 0.1, the minimum length at 10 mm, the maximum length at 200 mm, the number of tracks at 100,000. The result of the whole brain tractography is called the original connectome (Sporns et al., 2005; Pestilli et al., 2014).

#### LiFE optimization

LiFE is a global connectome evaluation method that uses the whole brain tractography to predict diffusion measurements (Pestilli et al., 2014). It is realized by solving the minimization problem as

(1) $$\begin{array}{l} {\text{argmin}}_{w_{f}}\sum_{v\in C}\sum_{\theta }{\left(M\left(\theta ,v\right)-\sum_{f\in v}w_{f}O_{f}\left(\theta \right)\right)}^{2},\\ \;w_{f}\geq 0 \end{array}$$

where *M*(θ, *v*) is the result of the measured diffusion signal subtracted by its mean, defined as

(2) $$\begin{array}{l} M\left(\theta ,v\right)=S_{v}\left(\theta ,b\right)-I_{v} \end{array}$$

*S*_*v*_(θ, *b*) is the measured diffusion signal in voxel of *v*, along the direction of θ and with the gradient strength *b*, *I*_*v*_ is the mean diffusion signal in the voxel. *O*_*f*_(θ) is the fiber-specific function and represents the diffusion signal around it mean.

(3) $$\begin{array}{l} O_{f}\left(\theta \right)=S_{0}\left(e^{-bA_{f}\left(\theta \right)}-\frac{1}{N_{\theta }}\sum_{\theta }e^{-bA_{f}\left(\theta \right)}\right) \end{array}$$

here *S*_0_ is the non-diffusion signal at a voxel and *A*_*f*_(θ) is the apparent diffusion coefficient in the direction θ for a single small segment of a fiber. Equation (1) is solved by non-negative linear least-squares algorithms. A set of weights for each fiber in the connectome *C*, *q*_*f*_, is the solution of this minimization problem. The root mean square error (r.m.s. error) between the prediction and the measured diffusion data can also be obtained.

#### Adjacency matrix construction

After LiFE optimization, only the fibers with positive weights remain to create the optimized connectome. The number of fibers in the optimized connectome may vary slightly for each individual in the range of 20,000–30,000. For each subject, we randomly select the same number of fibers as the optimized connectome to create a non-optimized connectome, and this kind of random selection is conducted 10 times to form a family of non-optimized connectomes. The aim is to ensure that the non-optimized and optimized connectomes are comparable.

For both the non-optimized and the optimized connectomes, we check each fiber and determine whether its two endpoints are located in two different brain regions *i* and *j*. If the fiber fulfills the condition, its index will be stored in the *I*(*i*,*j*), otherwise it will be discarded. With this *I*(*i*,*j*), one can easily calculate a 90 × 90 adjacency matrix *w*_*ij*_ according to different weighting methods, where the entry *w*_*ij*_ denotes the connection weight between the node *i* and *j*. Hence, one optimized network and one family of non-optimized networks (10 adjacency matrices) are constructed for each subject. For the comparison of the edges and for calculating the network measures, we use the mean of 10 adjacency matrices as the non-optimized network for an individual.

### Network weighting

For the optimized connectome, we have six kinds of weighting methods and weighted networks. The first one is the most straightforward, the fiber number weighted network (FN-N; Zhang et al., 2011).

(4) $$\begin{array}{l} w_{ij}^{FN}=M_{ij} \end{array}$$

where *M*_*ij*_ is the number of fibers connecting brain region *i* and *j*. The second one is the fiber density weighted network (FD-N), where $w_{ij}^{D}$, is defined as the number of fibers between two ROIs divided by the mean volume of the two ROIs (Buchanan et al., 2014).

(5) $$\begin{array}{l} w_{ij}^{FD}=\frac{{2M}_{ij}}{N_{i}+N_{j}} \end{array}$$

where *M*_*ij*_ denotes the number of fibers between nodes *i* and *j*, and *N*_*i*_ is the number of voxels in the ROI_*i*_. The third weighting is the fiber length, generating the fiber length weighted network (FL-N).

(6) $$\begin{array}{l} w_{ij}^{FL}=\frac{1}{M_{ij}}\sum_{s\in S_{ij}}l\left(s\right) \end{array}$$

where *S*_*ij*_ is the set of fibers connected node *i* and *j*, and *l*(*s*) is the length of the *s*th fiber in *S*_*ij*_. And the fourth one is the network weighted by the fiber density corrected by the fiber length (FDL-N; Hagmann et al., 2008).

(7) $$\begin{array}{l} w_{ij}^{FDL}=\frac{2}{N_{i}+N_{j}}\underset{s\in S_{ij}}{\sum }\frac{1}{l\left(s\right)} \end{array}$$

The fifth weighting method is unique for the optimized connectome, and it is defined as the fiber weight weighted network (FW-N).

(8) $$\begin{array}{l} w_{ij}^{FW}=\frac{1}{M_{ij}}\sum_{s\in S_{ij}}q_{f}\left(s\right) \end{array}$$

where *q*_*f*_(*s*) is the weight of the *s*th fiber in *S*_*ij*_ calculated from LiFE. The last one is the combination of the fiber number and weight, yielding the network weighted by the fiber contribution to predict the diffusion signals (FC-N).

(9) $$\begin{array}{l} w_{ij}^{FC}=\sum_{s\in S_{ij}}q_{f}\left(s\right) \end{array}$$

Finally six undirected positive weighted networks with setting self-connections zero can be obtained for the optimized network and the weighted matrices are denoted as *O*_$w_{ij}^{FN}$, ${O\text{\_}w}_{ij}^{FD}$, ${O\text{\_}w}_{ij}^{FL}$, ${O\text{\_}w}_{ij}^{FDL}$, $O\text{\_}w_{ij}^{FW}$, and ${O\text{\_}w}_{ij}^{FC}$. However, only four networks (*C*_$w_{ij}^{FN}$, ${C\text{\_}w}_{ij}^{FD}$, ${C\text{\_}w}_{ij}^{FL}$, ${C\text{\_}w}_{ij}^{FDL}$) can be constructed for the non-optimized network for lack of the information on the fiber weights.

### Network measures

Before calculating the network measures, the adjacency matrix will be normalized (scaled by the maximum element of the matrix) and be set with the same sparsity defined as

(10) $$\begin{array}{l} Sparsity=1.0-\frac{n_{a}}{n^{2}-n} \end{array}$$

where *n*_*a*_ is the number of available (or non-zero) edges. According to the sparsity setting, the top *n*_*a*_ edges in the list sorted by weight in descending order will be kept, and all the other edge weights will be set to zero. It is similar to the control of the edge density (or wiring cost, or edge number; (van den Heuvel and Sporns, 2011; Zhang et al., 2011). In the present study, we selected a sparsity range (0.75~0.95, step = 0.01), since there is no consensus with regard to the selection of a threshold.

For each weighted network, three commonly used nodal measures and three global network measures are calculated using the Brain Connectivity Toolbox (http://www.brain-connectivity-toolbox.net; Rubinov and Sporns, 2010). The three nodal measures that were used are the node strength (NS), the node efficiency (NE) and the node betweenness centrality (NBC). The node strength measures the sum of edge weights per node.

(11) $$\begin{array}{l} k_{i}=\sum_{j\in N}w_{ij} \end{array}$$

And the node efficiency is defined as

(12) $$\begin{array}{l} E_{nodal}\left(i\right)=\frac{1}{\text{n}-1}\sum_{j\in N,j\neq i}{d_{ij}}^{-1} \end{array}$$

where *d*_*ij*_ is the shortest path length between nodes *i* and *j*, can be calculated by the reciprocal of the edge weight. Lastly, the node betweenness centrality is

(13) $$\begin{array}{l} b_{i}=\frac{1}{\left(\text{n}-1\right)\left(\text{n}-2\right)}\sum_{\begin{array}{c} h,j\in N\\ h\neq j,h\neq i,j\neq i \end{array}}\frac{\rho_{hj}\left(i\right)}{\rho_{hj}} \end{array}$$

where ρ_*hj*_ is the number of shortest paths between *h* and *j*, and ρ_*hj*_(*i*) is the number of shortest paths between *h* and *j* that pass through *i*.

The three global measures are the characteristic path length, the mean clustering coefficient and small-worldness. The characteristic path length is to measure the function integration, and can be represented as

(14) $$\begin{array}{l} L=\frac{1}{n}\sum_{i\in N}\frac{\sum_{j\in N,\;j\neq i}d_{ij}}{n-1} \end{array}$$

The mean clustering coefficient is a measure of function segregation defined as

(15) $$\begin{array}{l} C=\frac{1}{n}\sum_{i\in N}\frac{2t_{i}}{k_{i}\left(k_{i}-1\right)} \end{array}$$

where *t*_*i*_ is the number of triangle around a node *i*. To know the balance between the segregation and integration, the small-worldness is proposed by Watts and Strogatz (1998).

(16) $$\begin{array}{l} \sigma =\frac{\gamma }{\lambda }=\frac{C∕C_{\text{rand}}}{L∕L_{\text{rand}}} \end{array}$$

where γ and λ are the normalized clustering coefficient and the normalized characteristic path length, respectively. *C*_rand_ and *L*_rand_ are the mean clustering coefficient and mean characteristic path length of 100 random networks with preserved degree distribution.

### Comparison and statistical analysis

Firstly, the fiber length distributions of the non-optimized and optimized connectomes are compared, because the connectomes are the bases of networks. Secondly, we do the edge-wise comparison to identify the edges with significantly different weights between the optimized group and the non-optimized group. If the optimized networks are considered to be more plausible, the edge presenting in the non-optimized network but being absent in the optimized network (i.e., ${C\text{\_}w}_{ij}^{FN}\neq 0$ and ${O\text{\_}w}_{ij}^{FN}=0$) will be determined as a false positive edge. Similarly the edge fulfilling the conditions $C\text{\_}w_{ij}^{FN}=0$ and ${O\text{\_}w}_{ij}^{FN}\neq 0$ will be a possible missing edge. Thirdly, we treat and compare the edges as a bag or collection where the interactions of edges are not taken into account (Craddock et al., 2013). Therefore, the correlation of two edge collections can be generated. Fourthly, for each node, the strength, efficiency and betweenness centrality will be compared between the optimized and non-optimized groups. Finally, the global network measures of the characteristic path length, the mean clustering coefficient and small-worldness of the optimized and non-optimized networks will be compared in the sparsity range of 0.75–0.95.

A non-parametric permutation test is used to determine whether the parameters of the optimized connectome group are significantly different with those of the non-optimized connectome group (Zhang et al., 2011; Xu et al., 2014). The parameters for comparisons include the fiber length distribution, the edge weight (FN, FD, FL, FDL, FW, and FC), three nodal network measures (*k*_*i*_, *E*_*nodal*_(*i*) and *b*_*i*_) and three global network measures (γ, λ, and σ). The non-parametric permutation test does not rely on the *t*-distribution, and estimates the null distribution by permutations of group labels. The number of permutations is set as 5000, which is a sufficiently large number. It *p* < 0.05, we reject the null hypothesis. For edge-wise multiple comparisons, we control the false discovery rate using the procedure as introduced by Benjamini and Hochberg (1995). Pearson's linear correlation coefficient are used to present the pair-wise linear correlation.

## Results

### Comparison of fiber length distributions

**Figure 2:**
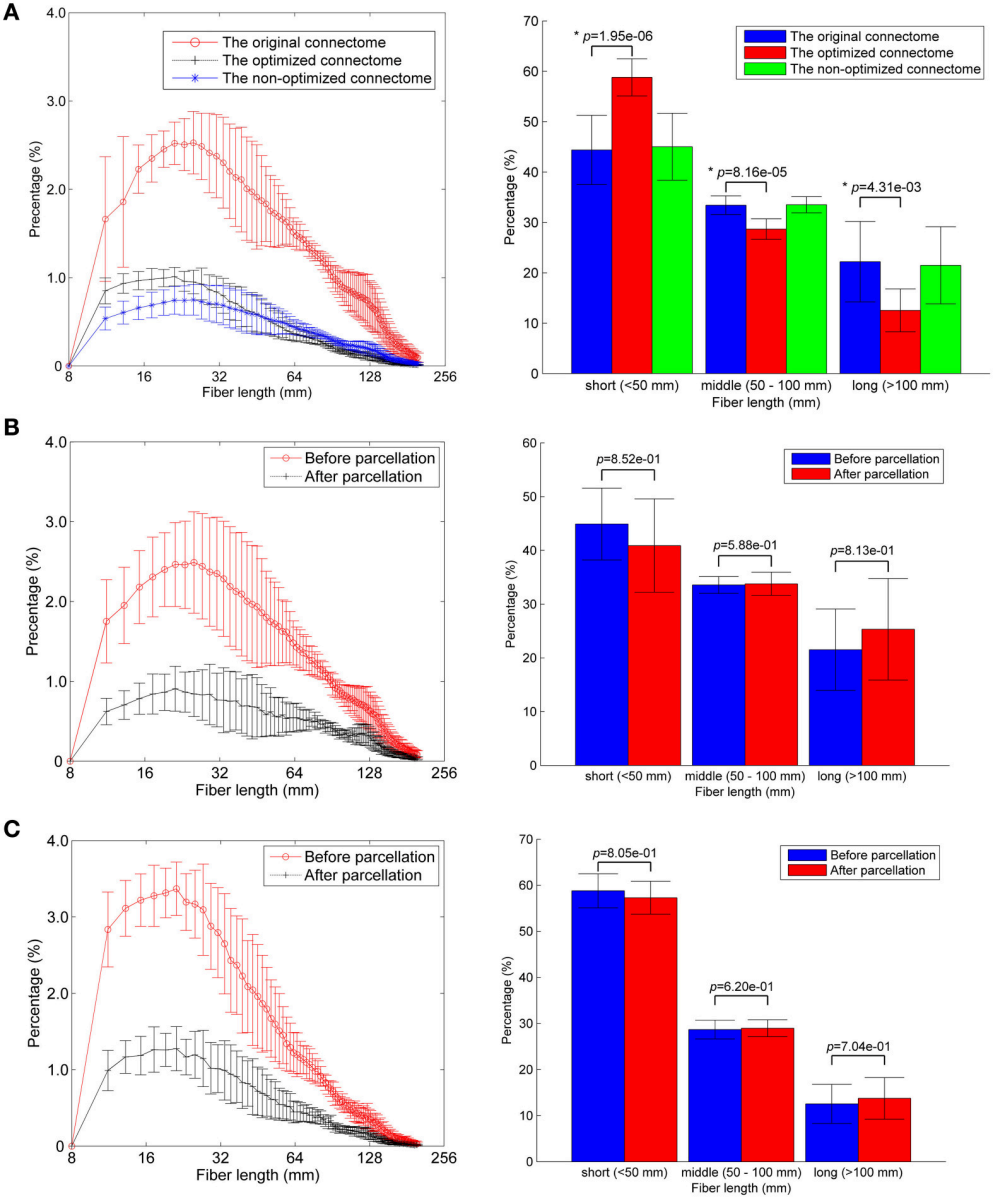
**Fiber length distributions**. **(A)** The fiber length distributions of the original connectome, the optimized connectome with LiFE, and the non-optimized connectome without LiFE. Left column is the histogram where the mean and standard deviation are plotted for nine healthy subjects. Right column indicates the percentages of short, middle, and long fibers. **(B)** The fiber length distribution of the non-optimized connectome before and after parcellation. **(C)** The fiber length distribution of the optimized connectome before and after parcellation.

About one third of the fibers in the original connectome have positive weights according to the LiFE algorithm, and are retained to form the optimized connectome, as shown in Figure 2A. The right part of the figure shows that the percentage of the short fibers (< 50 mm) in the optimized connectome (58.8 ± 3.7%) is significantly higher than that in the original connectome (44.4 ± 6.9%; permutation test, *p* < 0.05). It indicates that more short fibers are retained in the optimized connectome. Correspondingly, less middle (50–100 mm) and long fibers (>100 mm) exist in the optimized connectome, with the percentage of 28.7 and 12.5 %, respectively. The result is in agreement with previous finding that LiFE is pruning more the longer fibers (Pestilli et al., 2014).

The number of total fibers reduces with about 60% after parcellation for both the non-optimized connectome (Figure 2B) and the optimized connectome (Figure 2C). Brain parcellation using AAL90 actually deletes the fibers that do not connect any pair of atlas brain regions. Here, the selection criterion is very strict, i.e., only the fibers whose two endpoints are located in the different two regions in the AAL90 atlas can survive. The right part of the figures shows that no significant change occurs for the percentages of short, middle and long fibers between the results before and after parcellation (permutation test, *p* > 0.05). It indicates that the parcellation is non-selective for the fiber length, but removes the fibers that do not connect with any pair of brain regions.

### Optimized networks using different weighting methods

**Figure 3:**
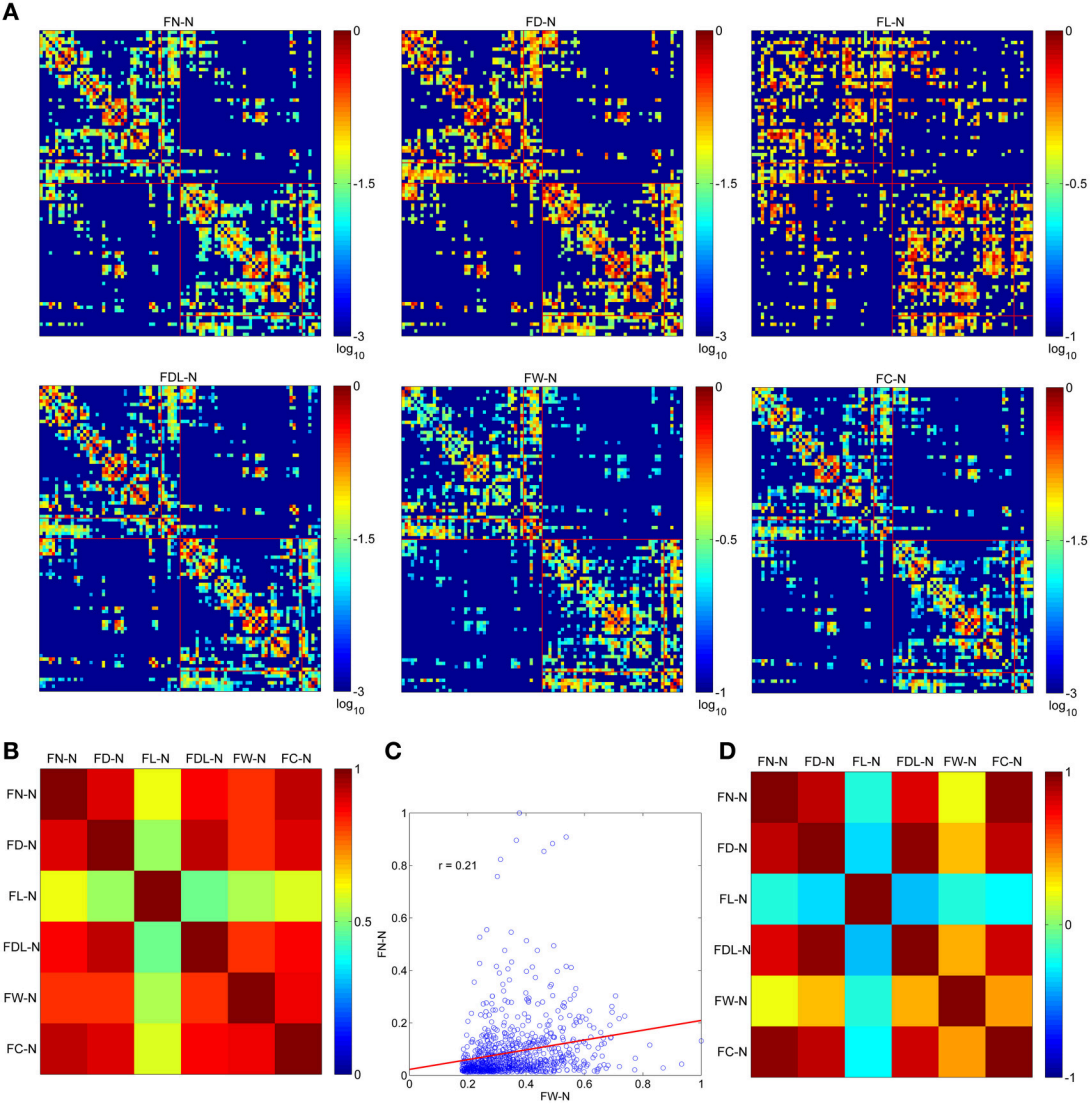
**The optimized networks weighted by six methods and their relationships**. **(A)** The weighted adjacency matrix (FN-N, the fiber number weighted network; FD-N, the fiber density weighted network; FL-N, the fiber length weighted network; FDL-N, the network weighted by the fiber density corrected by fiber length; FW-N, the fiber weight weighted network; FC-N, the network weighted by the fiber contribution to predict the diffusion signals). **(B)** The overlapping ratio of edges of two weighting methods. **(C)** The correlation of $w_{ij}^{FN}$ and $w_{ij}^{FW}$. **(D)** The correlation between each pair of weighting methods.

The optimized network can be presented by six weighting methods, as shown in Figure 3A. All of them are set at the same sparsity of 0.75 chosen arbitrarily. It is shown that the edges cluster densely along the main diagonal of the adjacency matrix for FN-N, FD-N, FDL-N, FW-N, and FC-N, but not for FL-N. This is in accordance with the earlier studies and the prior anatomical knowledge that the neighbor regions are densely connected by short distance fibers. The spatial pattern of edges in the FL-N appears to be more random and in contradiction with the expected pattern. The dynamic range of FL-N and FW-N is rather narrow, amounting to one order of magnitude, compared to FN-N and FD-N (three orders of magnitude).

The overlapping ratios of edges between different weighting methods are presented in Figure 3B. It is not surprising to observe that the overlap ratio between FL-N and the others is rather low, ranging from 0.48 to 0.61, as shown in Figure 3C. For the other weighting methods, the ratio varies from 0.82 (FDL-N and FW-N) to 0.93 (FN-N and FC-N).

However, the correlation coefficient between the edges of FN-N and FW-N is only 0.21, indicating different attributes of the network, as given in Figure 3D. The edges weights in FL-N are negatively correlated with the weights in other networks, and *r* varies from −0.38. to −0.20. It indicates that the edges with high fiber number and fiber weight tend to be short. FD-N, FDL-N, and FC-N are naturally and strongly correlated to FN-N (*r* = 0.85, 0.79, 0.94) because they are based on the fiber number. Moreover, one can refer to Supplementary Figure 1 for the adjacency matrices of the non-optimized networks with various weighting methods and their relationships.

### Edge related comparison

**Figure 4:**
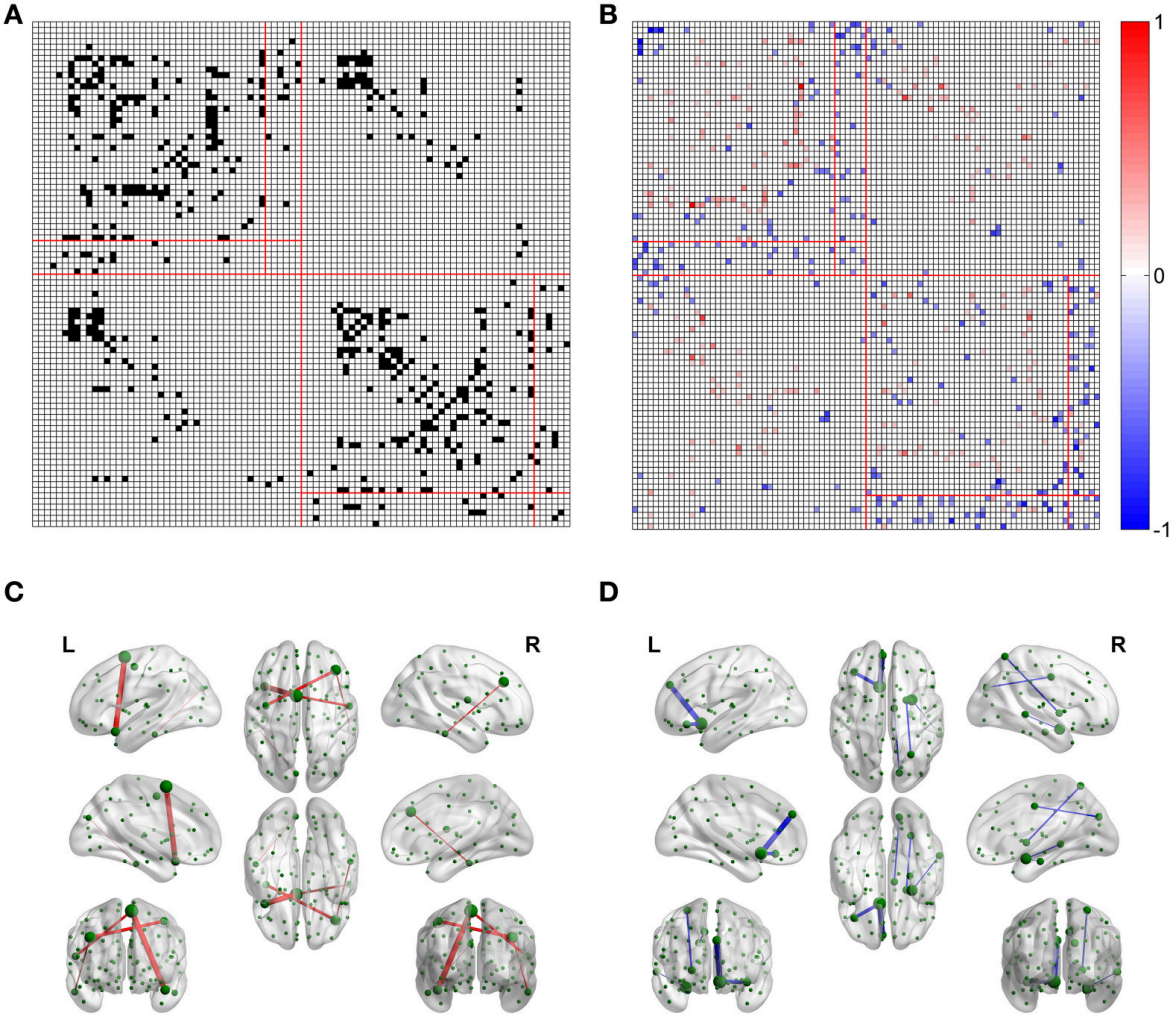
**Differences between the edges of the optimized and non-optimized networks weighted by the fiber number (FN-N). (A)** The difference between the weighted edges in the optimized and non-optimized networks at a sparsity of 0.75 (The edges with significant difference (*p* < 0.01) are shown in black). **(B)** False positive edges and possible missing edges at a sparsity of 0.75. The legend at right shows the normalized weights, and the colors of red and blue indicate the false positive edges and possible missing edges, respectively. **(C)** The false positive edges (Top 5). **(D)** The possible missing edges (Top 5).

For the FN-N with a sparsity of 0.75, 24.0% edges (480 of 2004) of the optimized network are significantly different from their counterparts in the non-optimized network (corrected *p* < 0.05), as given in Figure 4A. The spatial distribution of the significantly different edges are generally symmetrical between the left (254 edges) and right (226 edges) hemispheres. For the weighing methods of FD-N, FL-N, and FDL-N, similar results are observed (see the left part of Supplementary Figure 2).

The false positive edges and the possible missing edges can be determined through comparing the optimized and non-optimized networks, and their distributions are shown in Figure 4B for FN-N with a sparsity of 0.75. About 13.2% of the edges (264 of 2004) might be false positives, the same numbers of edges are thought to be the possible missing edges, indicating that the overlap ratio of the optimized and non-optimized matrices is about 73.6%. More than half (57.8%) possible missing edges are connected to the subcortical regions although there are only 12 subcortical regions. It may suggest that the optimized network emphasizes the fibers connecting the subcortical regions. In addition, the right part of Supplementary Figure 2 shows the situation of the weighting methods of FD-N, FL-N, and FDL-N.

Table 1 lists the Top 5 in the list of false positive edges sorted by the weight of FN and the Top 5 in the list of possible missing edges sorted by the weight of FN, while the spatial locations of these edges are shown in Figures 4C,D. It can be seen that two of the Top 5 false positive edges are commissural fibers, but all of the Top 5 possible missing edges are associational fascicles. It appears that the length of the Top 5 false positive edges is longer than that of the Top 5 possible missing edges, which may suggest that the number of long edges in the optimized network is diminished. However, the Table 1 highly depends on sparsity, because the edges with small $w_{ij}^{FN}$ are omitted.

**Table 1:** **The top 5 of false positive edges and possible missing edges**.

**Rank**	**Region A (Index and abbr.)**	**Region B (Index and abbr.)**	**$w_{ij}^{FN}$**	**$w_{ij}^{FD}$**	**$w_{ij}^{FL}$**	**$w_{ij}^{FDL}$**	**$w_{ij}^{FW}$**	**$w_{ij}^{FC}$**
**TOP 5 IN THE LIST OF FALSE POSITIVE EDGES SORTED BY THE WEIGHT OF FN**								
1	12, SMA.L	33, TPOsup.L	0.09	0.22	0.43	0.04	–	–
2	14, PreCG.L	53, MFG.R	0.07	0.07	0.59	0.01	–	–
3	12, SMA.L	60, ROL.R	0.07	0.15	0.54	0.03	–	–
4	53, MFG.R	77, SOG.R	0.05	0.05	0.72	0.01	–	–
5	26, CUN.L	32, ITG.L	0.05	0.09	0.67	0.01	–	–
**TOP 5 IN THE LIST OF POSSIBLE MISSING EDGES SORTED BY THE WEIGHT OF FN**								
1	2, OLF.L	11, SFGmed.L	0.04	0.07	0.31	0.02	0.20	0.01
2	76, MTG.L	86, AMYG.R	0.03	0.04	0.39	0.02	0.22	0.01
3	2, OLF.L	6, ORBinf.L	0.03	0.10	0.16	0.08	0.30	0.02
4	71, CUN.R	82, MCG.R	0.03	0.05	0.48	0.02	0.22	0.01
5	62, SPG.R	89, PAL.R	0.03	0.07	0.59	0.01	0.45	0.03

^*^For the false positive edges, the weights are determined according to the non-optimized networks, so that $w_{ij}^{FW}$ and $w_{ij}^{FC}$ do not exist. For the possible missing edges, the weights are determined according to the optimized networks.

**Figure 5:**
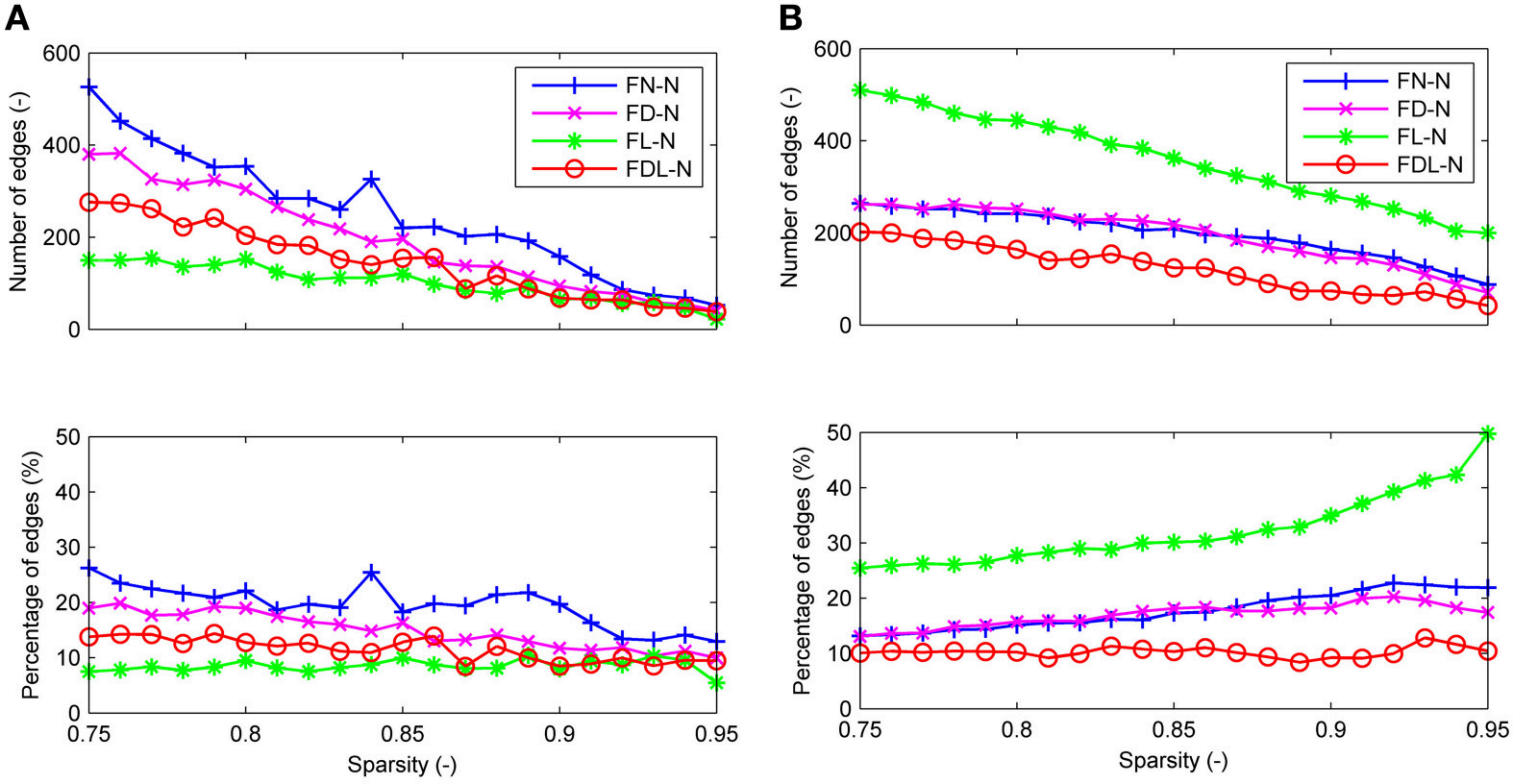
**The differences between the edges of the optimized and non-optimized networks weighted by various methods in the sparsity range of 0.75–0.95**. **(A)** The number and percentage of significantly different edges as a function of sparsity. **(B)** The number and percentage of false positive edges as a function of sparsity.

For all four weighting methods (FN-N, FD-N, FL-N, and FDL-N), the edges with significantly different weights in the non-optimized and optimized networks appear in the sparsity range of 0.75–0.95, but their number decreases gradually (Figure 5A). Even at the sparsity of 0.95, there are still 22 significantly different edges (5.5%) for FD-N, 52 significantly different edges (12.9%) for FN-N. The four weighting methods FN-N, FD-N, FDL-N, and FL-N are sorted in descending order by the number and percentage of the significantly different edges. The reason for the small number of different edges for FL-N might be the small dynamic range, while for FDL-N, the reason may lie in the combination of weighing methods.

Similarly the number of false positive edges drops with the increase of sparsity for all the weighting methods (Figure 5B). However, the percentages nearly remain constant (about 10%) for FDL-N, and increase for FN-N, FD-N, and FL-N. The reason why the number of false positive edges decreases while the percentage increases can be explained by the decreasing total number of edges in the optimized network with sparsity. If the total number drops faster than the number of false positive edges, the percentage will increase. Moreover, it is observed that the percentage of the false positive edge reaches 49.8% at the sparsity of 0.95 for FL-N, indicating that almost no non-zero edges exist in the optimized and the non-optimized networks simultaneously. It also shows that the distribution of edge lengths is quite different for the optimized and non-optimized networks.

**Figure 6:**
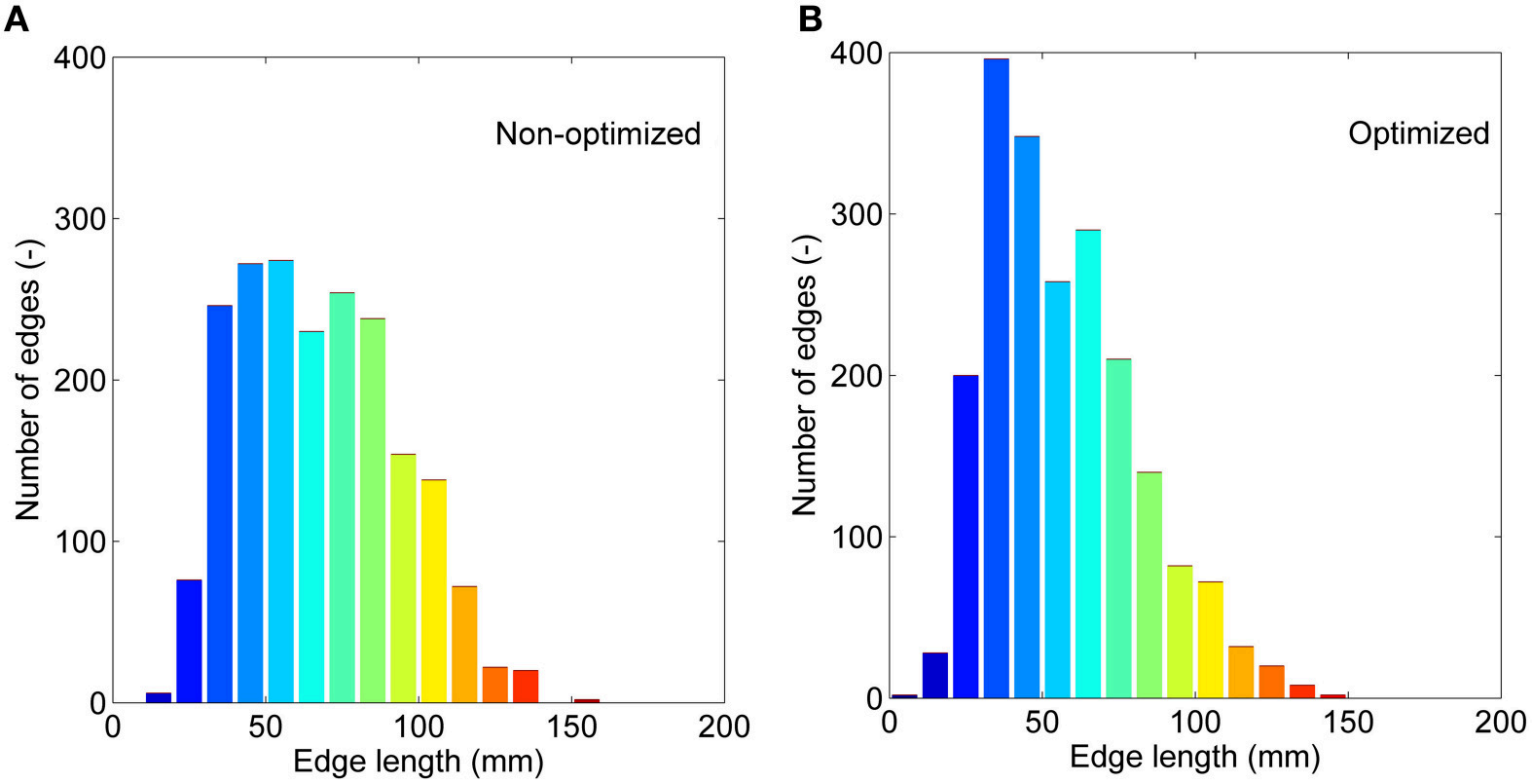
**The edge length (averaged for nine subjects) distributions. (A)** The non-optimized network. **(B)** The optimized network.

If we treat all the edges as a bag or collection and don't consider their interactions, we can compare the non-optimized and optimized networks based on: (1) the correlations between edge weights, and (2) the edge length distribution. Significant correlations are found for all four weighting methods (see Supplementary Figure 3). The weighting method of FL presents the lowest *r* of 0.58, and FDL method shows the highest *r* of 0.98. It further demonstrates that the optimized network has edges with different length with those of the non-optimized network. The correction by the fiber length can decrease this difference. The edge length distributions are given in Figure 6 for the non-optimized and optimized networks. A heavy-tailed distribution can be observed for both networks, which agrees well with the reports of earlier studies (Gigandet et al., 2013; Crossley et al., 2014). Note, however, that the distributions are quite different. More short edges, epically in the range of 20–50 mm, exist in the optimized network, while the number of edges with the length of 80–120 mm correspondingly drops. It is thought to be the consequence of more short fibers in the optimized connectome.

### Node related comparison

**Figure 7:**
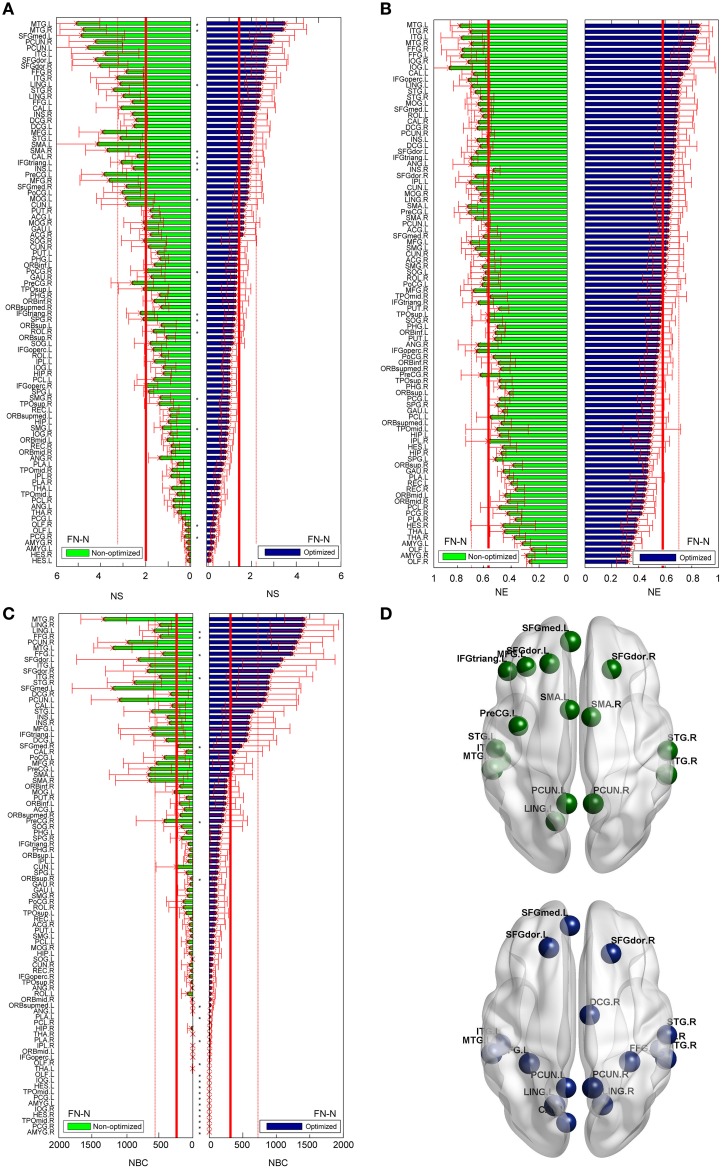
**Differences of the nodal measures between the optimized and non-optimized networks weighted by the fiber number (FN-N)**. Here^*^ indicates there is a significantly difference between the measures from the non-optimized network and the optimized network (*p* < 0.05). The nodes are ordered according to the nodal measures of the optimized network. The vertical thin and bold lines indicate the *mean* and *mean* + *SD* of the measures of all nodes. **(A)** The node strength (NS). **(B)** The node efficiency (NE). **(C)** The node betweenness centrality (NBC). **(D)** The nodes with high NBC (>*mean* + *SD*; The first row is for the non-optimized network and the second row is for the optimized network).

The differences between the nodal measures of the optimized and non-optimized networks are not as large as for the edge related comparisons. For FN-N, some nodes (16 of 90) show significantly different strength (Figure 7A). No node is observed to have significantly different node efficiency (Figure 7B), and several nodes with high betweenness centrality (e.g., LING.L, FFG.R, FFG.L, and STG.R) present significant differences (Figure 7C). It is noted that the sequences of the node strength, efficiency and betweenness centrality have changed, which may influence the determination of hubs (Hagmann et al., 2008). Figure 7D shows the nodes of *b*_*i*_ > *mean* + *SD*, where *mean* and *SD* is the average and standard deviation of nodal betweenness centrality over all nodes of the network, respectively. Sixteen nodes meet the requirement for both the non-optimized and optimized networks, 10 nodes are the same. They are STGmed.L, SFGdor.L, ITG.L, MTG.L, PCUN.L, LING.L, SFGdor.R, PCUN.R, STG.R, MTG.R.

**Figure 8:**
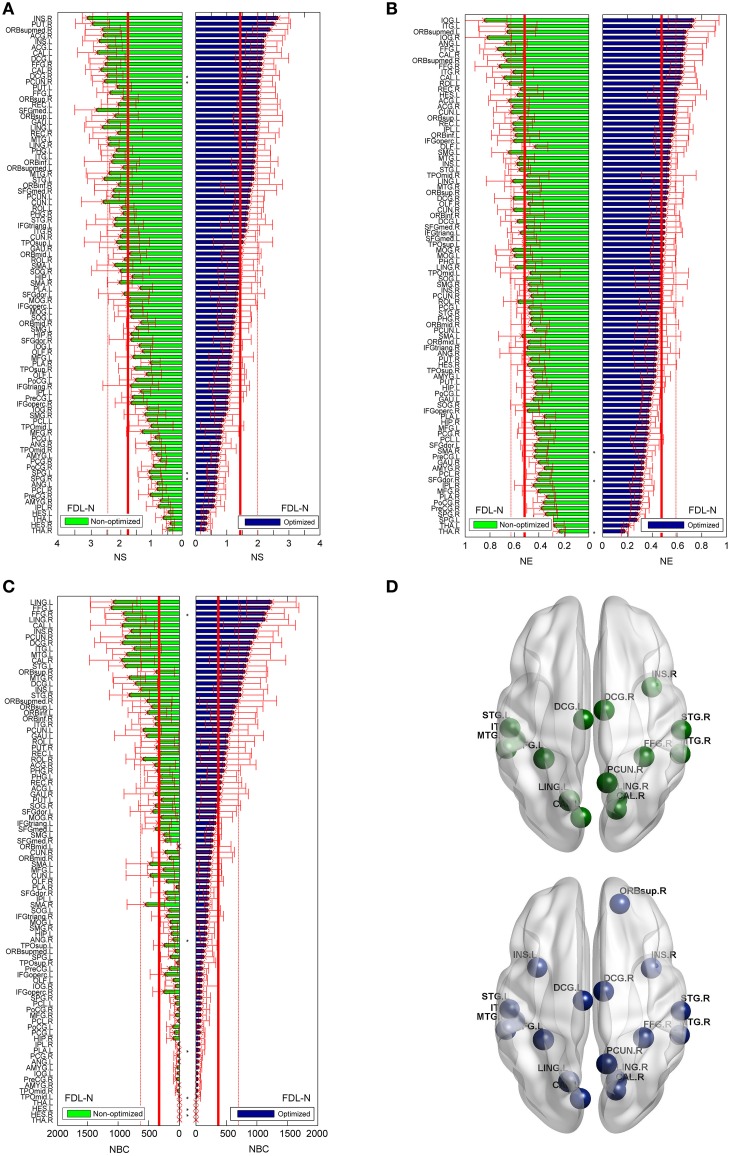
**Differences of the nodal measures between the optimized and non-optimized networks weighted by the fiber density corrected by the fiber length (FDL-N)**. Here^*^ indicates there is a significantly difference between the measures from the non-optimized network and the optimized network (*p* < 0.05). The nodes are ordered according to the nodal measures of the optimized network. The vertical thin and bold lines indicate the *mean* and *mean* + *SD* of the measures of all nodes. **(A)** The node strength (NS). **(B)** The node efficiency (NE). **(C)** The node betweenness centrality (NBC). **(D)** The nodes with high NBC (>*mean* + *SD*; The first row is for the non-optimized network and the second row is for the optimized network).

As shown in Figure 8, the trends of nodal measures of FDL-N are similar to FN-N. Because FDL-N is the combination of the fiber density, number and length, the differences between the non-optimized and optimized network are smaller than FN-N at the node level. There are 15 nodes meeting the condition of *b*_*i*_ > *mean* + *SD* in the non-optimized network, and all of them appear in the 17 nodes in the optimized networks.

For the FD-N and FL-N, one can refer to Supplementary Figures 4, 5 to know the node-level comparison. For FW-N and FC-N, only the results of the optimized network are given in Supplementary Figure 6 because these two weighting methods are not available for the non-optimized network.

Moreover, three nodal measures show different variations within 90 nodes: *b*_*i*_ is the largest, *E*_*nodal*_(*i*) is the second and *k*_*i*_ is the smallest. It is indicated that each measure denotes one distinct attribute of the node, and they can be combined to define the possible hubs. Comparison across weighting methods presents that the variations of three measures are small for FL-N and FW-N. Especially for the node efficiency, all the nodes seem to have nearly equal values.

### Global network measures related comparison

**Figure 9:**
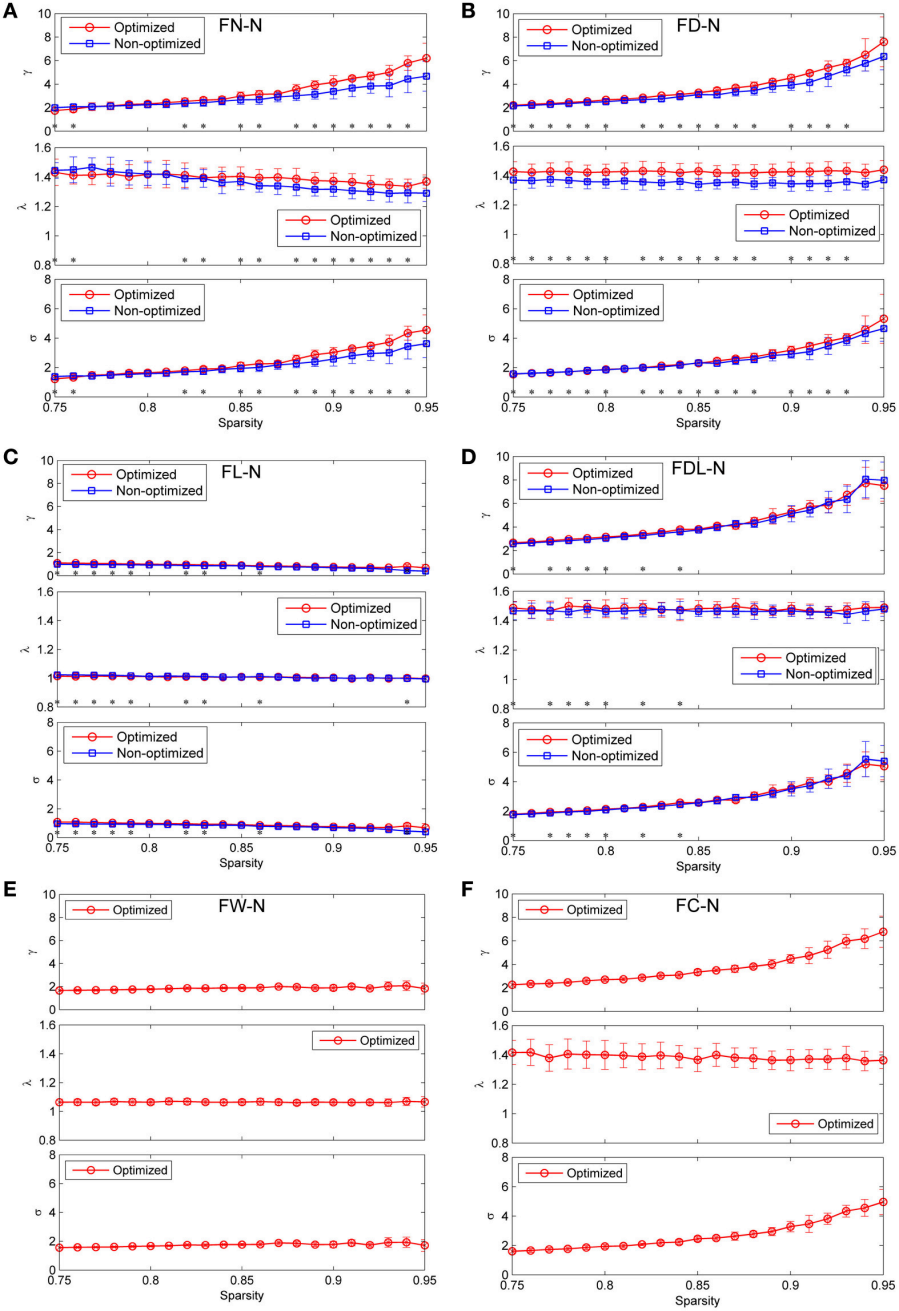
**Comparison the global network measures between the optimized and non-optimized networks (the normalized mean characteristic path length, the normalized mean clustering coefficient and the small-worldness)**. **(A)** The networks weighted by the fiber number (FN-N). **(B)** The networks weighted by the fiber density (FD-N). **(C)** The networks weighted by the fiber length (FL-N). **(D)** The networks weighted by the fiber density corrected by the fiber length (FDL-N). **(E)** The network weighted by the fiber weight (FW-N). Because the weighting method of FW does not exist for the non-optimized network, only the optimized network is shown. **(F)** The network weighted by the fiber contribution (FC-N). Because the weighting method of FC does not exist for the non-optimized network, only the optimized network is shown.

The global measures of γ, λ, and σ are presented as function of the sparsity in Figure 9 for both the non-optimized and optimized networks. It can be observed that FL-N and FW-N demonstrate remarkably different characteristics from FN-N, FD-N, FDL-N, and FC-N. For FL-N, σ is always smaller than 1.0 in the studied sparsity range, decreasing from 0.96 to 0.40 while the sparsity rises from 0.75 to 0.95. Since λ is nearly equal to the constant of 1.0, the variation of γ is the same as σ. It is suggested that FL-N is not a small-worldness network but a random network. For FW-N, γ and σ slightly increase with the sparsity and range from 1.56 to 1.93. These abnormal characteristics indicate that the fiber length and the fiber weight are not suitable to be independent weighting methods.

For the FN-N, FD-N, FDL-N, and FC-N, one can see two common features: (1) High γ and short λ lead to the small-worldness attribute (σ >1.0 and ranges from 1.22 to 5.53) in the studied sparsity range. (2) Whereas γ increases with the sparsity rapidly, λ deceases slightly or remains constant, which leads to an increasing σ. These common features may originate from the fact that FD-N, FDL-N, and FC-N are based on FN-N by various kinds of corrections.

There are some differences between the global network measures of the non-optimized and optimized networks across the weighting methods, though the measures are agglomerative in nature. For FN-N, the optimized network owns higher γ and λ than the non-optimized networks when the sparsity is larger than 0.87 (Figure 9A). It is because more short edges lead to high γ and function segregation, while less long edges lead to high λ and high cost of function integration. The increase of γ exceeds that of λ, which results in higher σ for the optimized network. It can be further inferred that the edges with high weights who survive at higher sparsity are quite different for the non-optimized and optimized networks. The results of γ, λ, and σ for FD-N seem to be similar to FN-N, as shown in Figure 9B. While for the FDL-N (Figure 9C), significant differences are observed only at low sparsity (< 0.85). It suggests that the edges with low weights are different for the two networks, the correction of the fiber lengths has diminished the difference.

### Application to HCP datasets

**Figure 10:**
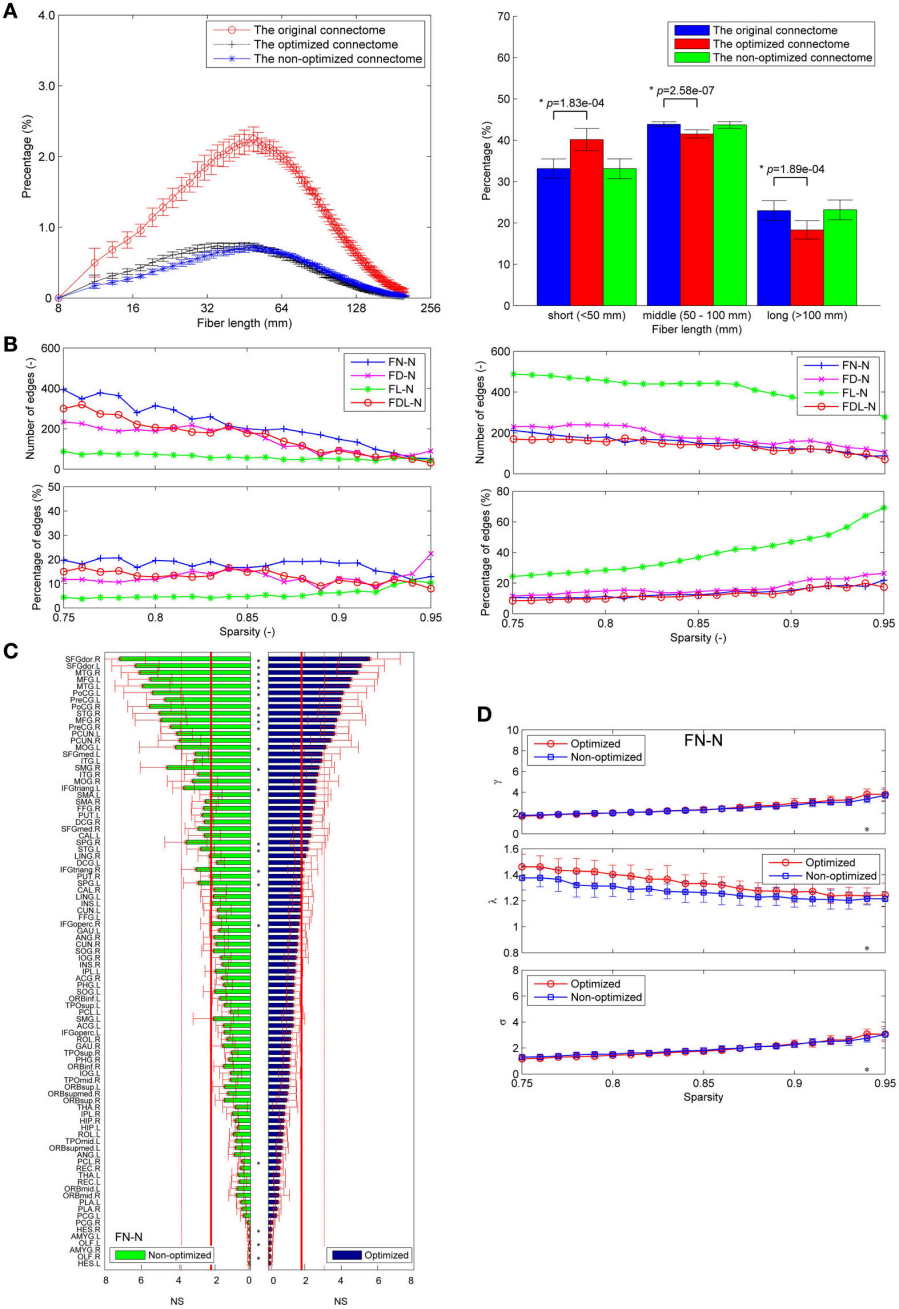
**Application to HCP dataset**. **(A)** The fiber length distributions of the original connectome, the optimized connectome with LiFE and the non-optimized connectome without LiFE. **(B)** The differences between the edges of the optimized and non-optimized networks weighted by various methods in the sparsity range of 0.75–0.95. Left column indicates the number and percentage of significantly different edges as a function of sparsity. Right column indicates the number and percentage of false positive edges as a function of sparsity. **(C)** Differences of the node strength (NS) between the optimized and non-optimized networks weighted by the fiber number (FN-N). **(D)** Comparison the global network measures between the optimized and non-optimized networks (the normalized mean characteristic path length, the normalized mean clustering coefficient, and the small-worldness) for the networks weighted by the fiber number (FN-N).

Although the diffusion MRI scanning protocols are different, most results obtained using HCP datasets are similar with those obtained from our own dataset. Firstly, more short fibers are found in the optimized connectome, as shown in Figure 10A. Secondly, there are 5.0~20% edges who have significantly different weights in the non-optimized and optimized networks (Figure 10B). More than 10% edges are considered to be the false positives. Thirdly, amount of nodes (22 out of 90) show different node strength for the non-optimized and optimized FN-N at a sparsity of 0.75 (Figure 10C). This situation occurs for the node centrality betweenness and other weighing schemes. Supplementary Figures 7–9 present more details about the fiber length distribution, edge differences and node measure differences for HCP datasets, respectively.

However, the fiber length distributions of the connectome from HCP dataset are different with those from our own data, i.e., the former owns more fibers with middle length (50-100 mm). It may be resulted from the different scanning protocols (64 and 32 directions), and agrees with the study by Gigandet et al. (2013). More fibers with middle length also make the effect of LiFE on the global network measures obtained from HCP dataset (Figure 10D) smaller than that on the measures obtained from the our own scanned data (Figure 9A).

## Discussion

In the present study, the LiFE method has been applied to create an optimized connectome through the elimination of redundant and nonexistent fibers. Subsequently the optimized networks are constructed with six kinds of weighting schemes. The non-optimized and the optimized network are systematically compared in order to clarify the effect of LiFE optimization on a structural brain network. The result of companions shows that there are some significant differences in the fiber length distribution, the edge weights, the nodal network measures, and the global network measures between the results with and without LiFE optimization.

### Important role of LiFE optimization

The important role of LiFE optimization used here is to remove the redundant and nonexistent fibers from the whole brain tractography and generate the optimized connectome. To our knowledge, no specific and systemic study has been conducted earlier to clarify the effect of LiFE optimization on the structural brain network. Some other methods which can avoid or reduce the occurrence of false positive fibers before or during tracking are also very crucial (Parker et al., 2003; Sotiropoulos et al., 2010; Lienhard et al., 2011; Tax et al., 2014). However, their principles are quite different from LiFE optimization. LiFE identifies and removes the false positive fibers for the global benefit, i.e., the minimum r.m.s. error between the prediction using the whole brain tractography and the measured dMRI signals.

Our results show that LiFE optimization has an important impact on the connectome. Relatively more short fibers are selected from the original connectome by LiFE optimization (Figure 2), which indicates that the selection is correlated to the fiber length. Hence, the fiber length distribution has been changed. It is in accordance with anatomical histology which shows even more short fibers (< 10 mm; Braitenberg and Schuz, 1998) and neural development mechanism promoting short wiring (Sporns, 2010). Pestilli et al. (2014) reported that the optimized connectome includes more short (< 50 mm) than long (>100 mm) fibers. They assumed, without systematic assessment, that the reduction of long and short fibers was approximately equal.

It should be noted that one must be very careful while interpreting the “optimized connectome.” Here, the optimized connectome only means that the retained fibers with positive weights can predict the measured diffusion data well, even with smaller r.m.s. error than the prediction by all the fibers with the same weight of one and the test-retest data (Pestilli et al., 2014). It is not related to the “wiring minimization,” i.e., the efficiency or economy of neural wiring described by Bullmore and Sporns (2012). In this paper, the meaning of the optimized network is that it is derived from the connectome after LiFE optimization.

### Optimized networks with various weighting schemes

Up to now, several alternative weighting methods have been proposed and implemented in earlier studies of the structural brain network. FN is the most straightforward and basic weighting methods (Gong et al., 2009). The fiber density (Bassett et al., 2011; Cheng et al., 2012) and the fiber density corrected by the fiber length (Hagmann et al., 2008; Zhang et al., 2011; Li et al., 2012) are the other two commonly used weighting methods. The motivation of normalization by fiber length is to remove the intrinsic linear bias toward longer fibers of deterministic tractography (Hagmann et al., 2008). However, it will be difficult to correct for this effect when using probabilistic tractography (Li et al., 2012). Moreover, the independent attributes of the edges can also be another category of weighting schemes. One of the important examples is the fiber length (Crossley et al., 2014). White matter microstructural properties, such as the fractional anisotropy (FA) and the mean diffusivity (MD), the intra-cellular volume fraction (ICVF), and the scalar-valued orientation dispersion index (ODI), have also been used to weight the connectivity (Buchanan et al., 2014; Lemkaddem et al., 2014).

Besides the four commonly used weighting methods (FN, FL, FD, and FDL), in the present study new weighting methods are generated: the fiber weight (FW), and the combination of the fiber weight and fiber number (the fiber contribution to predict the diffusion signals, FC). These two new weighting methods only exist for the optimized network, while their specific roles need further explorations.

We found that FW and FL have a dynamic range of an order of magnitude, which is quite narrow compared to the weighting method of FN (three orders of magnitude; Figure 3A). It suggests that FW-N and FL-N might have low sensitivity in depicting the edges. For FL-N, the spatial pattern of edges (Figure 3A), the correlation and overlap ratio of the edges (Figures 3B,C), the variations of nodal measures (Supplementary Figure 4), and the global network measures (Figure 9) are quite different compared to the other weighting methods. Even the robust small-worldness attribute is not conserved. All the above findings indicate that FL is not a suitable weighting method to explore the topological properties of the structural brain network. We also found that FW is similar to FL with regard to the narrow dynamic range, low overlap ratio, and correlation coefficient and small variations of nodal measures. Though σ is larger than 1.0, the variation with sparsity is much smaller than the other weighting methods (Figure 9). It is suggested that FW should be used carefully as an independent weighing method. For the weighting method of the fractional anisotropy (FA), similar results are reported by Zhang et al. (2011).

Our results indicate that FN is an excellent weighting method with three orders of magnitudes which is enough to precisely represent the subtle differences between edges. The combinations of FN and FA, FW show similar great properties (Li et al., 2009; Zhang et al., 2011). Another possible approach to utilize FA, FW, and FL is to multiply them with the binary mask determined through setting a sparsity threshold to the FN weighted matrix. Hence FA, FW, and FL of each edge can be assessed. For instance, the fat-tailed edge length distribution is observed (Gigandet et al., 2013; Crossley et al., 2014). FD and FDL are also derived from the FN. The combination and correction will increase the reproducibility while decreasing the sensitivity (Cheng et al., 2012; Buchanan et al., 2014), which explains why the differences between the non-optimized and optimized networks weighted by FN are larger than those of other weighting methods.

### Impact of LiFE optimization

The impact of LiFE optimization on the network measures is profound. The impact apparently originates from the fact that LiFE has changed the connectome (the basis of the network) with regard to the fiber length distribution.

The LiFE optimization influences the weight of individual edges and the edge spatial distributions. The influence on the edge weight leads to some edges, with a percentage as high as 24.0%, owning significantly different weights from their counterparts in the non-optimized network. Because the edges with small weights may be omitted through setting a fixed sparsity value, the influence of LiFE optimization on the edge weight expands to the edge spatial distribution and results in false positive and possible missing edges. It should be noted that LiFE optimization only can reduce the false positive fibers, but is not able to supply possible missing fibers (Pestilli et al., 2014). However, we can define the possible missing edge if it satisfies the requirement of ${O\text{\_}w}_{ij}^{FN}\neq 0$ and ${C\text{\_}w}_{ij}^{FN}=0$. Therefore, currently, it is of top priority to provide evidence for the occurrence of the false positives and possible missing edges, to build up a reliable backbone of structural brain network as a comparable reference by using widely accepted brain parcellation (e.g., AAL), data of a large population of healthy controls and advance dMRI and tractography techniques (Hagmann et al., 2008; Gong et al., 2009).

The nodal measures (NS and NBC) and node sequence are also affected for some nodes by LiFE optimization. The observation of high node betweenness centrality at STGmed.L, SFGdor.L, ITG.L, MTG.L, PCUN.L, LING.L, SFGdor.R, PCUN.R, STG.R, MTG.R. in general is in accordance with the previous studies (Hagmann et al., 2008; Gong et al., 2009). The changes of the sequence of node strength, efficiency and betweenness centrality will influence the hub identification, because hubs are thought to be highly connected and highly central (Sporns et al., 2007). As an example, the precuneus has been shown to be the hub of functional connectivity and with the highest metabolic rate (Buckner et al., 2009). However, it is beyond the scope of this paper to discuss hubs and modularity, so one can refer the related contents presented by Sporns (2010).

Even the robust and highly agglomerative global measures are changed as well, although the topological properties such as the small-worldness remain robustly. For FN-N, more short fibers results in more short edges and less long edges, which further leads to the high γ and long path length (high λ). Long path length infers low efficiency, a measure denoting the capacity to facilitate the information exchange within the network (Latora and Marchiori, 2001).

### Limitations and future directions

In this study, we tested the effect of LiFE optimization on the connectome generated through a CSD-based probabilistic algorithm. Actually one of the great features of LiFE is that it can be used to the whole brain tractography generated by any algorithm. The reason we apply the CSD-based probabilistic algorithm here is because of the great CSD features (Bastiani et al., 2012; Tournier et al., 2012; Wilkins et al., 2015), such as good fiber detection rates. In the future, different estimation models (e.g., DSI Cammoun et al., 2012) or multi-shell CSD (Jeurissen et al., 2014) and tractography algorithms (local or global) can be investigated (Bassett et al., 2011). Similarly, the multi scale studies using fine parcellations can be added (Hagmann et al., 2008; Zalesky et al., 2010; Bassett et al., 2011). Finally, our study was limited to a cohort of nine subjects and part data from the HCP dataset. More subjects can further increase the statistical power and lead to more reliable results.

In future, the role of LiFE optimization can be further developed to fully utilize its feature that the false positive fibers can be removed effectively, but the possible missing fibers are unable to be supplied. With LiFE optimization, actually one closed prediction-evaluation loop has been created. We can iterate this loop with adding new fibers continuously and ensure r.m.s. error decreases to a satisfied low value. One can also combine different ODF estimation models and tractography algorithms to generate the original connectome because DTI-based tractography is reported to outperform other advance techniques while tracking the short U-fibers (Rodrigues et al., 2013). Moreover, to set liberal termination criteria for the tracking algorithm is beneficial to avoid possible missing fibers.

The creation of a more reasonable and accurate structural brain network that was established in this study is important for further research, especially for studies on coupling and decoupling of structural and functional brain connectivity (Skudlarski et al., 2008; Honey et al., 2009; Zhang et al., 2011; Reijmer et al., 2015). Using a structural brain network as an anatomical constraint, a large-scale network of global brain dynamics can be derived to analyze the link between the anatomical structure, neural network dynamics and resting-state and task-evoked functional connectivity (Fontanini and Katz, 2008; Deco et al., 2013; Arsiwalla et al., 2015). These studies will eventually help to reveal the intrinsic mapping of structure and function of the brain (Pessoa, 2014).

The non-optimized and optimized networks were compared with regard to the edges, the nodal network measures, and the global network measures. However, high sensitivity is required to be able to identify significant difference between the normal brain networks and the ones related to neurological disorders (Liu et al., 2012; Besson et al., 2014). One approach named network-based statistic (NBS; Zalesky et al., 2010) gains statistic power through evaluating the null hypothesis at the sub-networks level rather than at edge-pair level independently, which makes it more sensitive to identify some statistically significant disturbances. For the node related comparison, the connectivity fingerprint of each node can be built up and compared just like the network functional fingerprints (Anderson et al., 2013; Pessoa, 2014). Combined with NBS and the connectivity fingerprints, the methods of comparing networks presented in this study might be the paradigm for the future to identify the differences between the normal brain networks and the networks related to neurological disorders.

## Conclusions

A step of eliminating redundant and nonexistent fibers from the whole brain tractography by LiFE is inserted into the pipeline of constructing a structural brain network and used to generate an optimized network. The optimized networks have been presented in multiple weighting methods (FN, FD, FL, FDL, FW, and FC), each revealing different attributes of the network. It was shown that LiFE optimization has a profound impact on the connectome and networks. More short fibers are retained in the optimized connectome. Some edges show significantly different weights after optimization, and some false positive and possible missing edges are observed. Also the network measures of node strength, node efficiency, and node betweenness centrality were altered after optimization, which influences the sorting of the nodes and the determination of hubs. Even the robust and highly agglomerative global measures such as the normalized clustering coefficient, the normalized characteristic path length and the small-worldness changed as well after LiFE optimization. Therefore, LiFE optimization is considered to be a critical step for constructing more reasonable and more accurate structural brain networks.

## Author contributions

SQ, SM, PO conceived, designed and performed the experiments together. SQ drafted the work and analyzed the data, SM, BT, KN interpreted part of data and revised the manuscript critically. All authors read and approved the manuscript.

### Conflict of interest statement

The authors declare that the research was conducted in the absence of any commercial or financial relationships that could be construed as a potential conflict of interest.
