# Supplementary material for: Structural Brain Network: What is the Effect of LiFE Optimization of Whole Brain Tractography?
Source: Front Comput Neurosci. 2016 Feb 16;10:12. doi: 10.3389/fncom.2016.00012 (PMC4754446; doi:10.3389/fncom.2016.00012)
Supplement: Supplementary file 1 [file Table1.DOCX]

***Supplementary Material***

**Structural brain network: What is the effect of the LiFE optimization of whole brain tractography?**

**Shouliang Qi^1,3,4^*, Stephan Meesters^2,3^, Klaas Nicolay^4^, Bart M. ter Haar Romeny^1,4^, Pauly Ossenblok^3,4^**

^1^Sino-Dutch Biomedical and Information Engineering School, Northeastern University, Shenyang, China

^2^Department of Mathematics & Computer Science, Eindhoven University of Technology, Eindhoven, the Netherlands

^3^Academic Center for Epileptology Kempenhaeghe & Maastricht UMC+, Heeze, the Netherlands

^4^Department of Biomedical Engineering, Eindhoven University of Technology, Eindhoven, the Netherlands

*** Correspondence:** Shouliang Qi, Sino-Dutch Biomedical and Information Engineering School, Northeastern University, Zhihui Street 500, Shenyang, 110167, China

qisl@bmie.neu.edu.cn

1. **Supplementary Tables**

**Supplementary Table 1. Abbreviations for the brain regions used in this study, according to Achard et al. (2006).** There are 90 regions in AAL atlas totally. The indexes of 1-45 are for the left hemisphere, and the others are for the right.

| **Index** | **Regions** | **Abbr.** |
| --- | --- | --- |
| 1 | Gyrus Rectus | REC |
| 2 | Olfactory Cortex | OLF |
| 3 | Supeiror frontal gyrus, orbital part | ORBsup |
| 4 | Superior frontal gyrus, medial orbital | ORBsupmed |
| 5 | Middle frontal gyrus orbital part | ORBmid |
| 6 | Inferior frontal gyrus, orbital part | ORBinf |
| 7 | Superior frontal gyrus, dorsolateral | SFGdor |
| 8 | Middle frontal gyrus | MFG |
| 9 | Inferior frontal gyrus, opercular part | IFGoperc |
| 10 | Inferior frontal gyrus, triangular part | IFGtriang |
| 11 | Superior frontal gyrus, medial | SFGmed |
| 12 | Supplementary motor area | SMA |
| 13 | Paracentral lobule | PCL |
| 14 | Precentral gyrus | PreCG |
| 15 | Rolandic operculum | ROL |
| 16 | Postcentral gyrus | PoCG |
| 17 | Superior parietal gyrus | SPG |
| 18 | Inferior parietal, but supramarginal and angular gyri | IPL |
| 19 | Supramarginal gyrus | SMG |
| 20 | Angular gyrus | ANG |
| 21 | Precuneus | PCUN |
| 22 | Superior occipital gyrus | SOG |
| 23 | Middle occipital gyrus | MOG |
| 24 | Inferior occipital gyrus | IOG |
| 25 | Calcarine fissure and surrounding cortex | CAL |
| 26 | Cuneus | CUN |
| 27 | Lingual gyrus | LING |
| 28 | Fusiform gyrus | FFG |
| 29 | Heschl gyrus | HES |
| 30 | Superior temporal gyrus | STG |
| 31 | Middle temporal gyrus | MTG |
| 32 | Inferior temporal gyrus | ITG |
| 33 | Temporal pole: superior temporal gyrus | TPOsup |
| 34 | Temporal pole: middle temporal gyrus | TPOmid |
| 35 | Parahippocampal gyrus | PHG |
| 36 | Anterior cingulate and paracingulate gyri | ACG |
| 37 | Median cingulate and paracingulate gyri | MCG |
| 38 | Posterior cingulate gyrus | PCG |
| 39 | Insula | INS |
| 40 | Hippocampus | HI |
| 41 | Amygdala | AMYG |
| 42 | Caudate nucleus | CAU |
| 43 | Lenticular nucleus, putamen | PUT |
| 44 | Lenticular nucleus, pallidum | PAL |
| 45 | Thalamus | THA |
