# Supplementary material for: Structural Brain Network: What is the Effect of LiFE Optimization of Whole Brain Tractography?
Source: Front Comput Neurosci. 2016 Feb 16;10:12. doi: 10.3389/fncom.2016.00012 (PMC4754446; doi:10.3389/fncom.2016.00012)
Supplement: Supplementary file 2 [file Image1.PDF]

frontiers

Supplementary Material

Structural brain network: What is the effect of the LiFE optimization of whole brain tractography?

Shouliang Qi[1,3,4]*, Stephan Meesters[2,3], Klaas Nicolay[4], Bart M. ter Haar Romeny[1,4], Pauly Ossenblok[3,4]

[1]Sino-Dutch Biomedical and Information Engineering School, Northeastern University, Shenyang, China
[2]Department of Mathematics & Computer Science, Eindhoven University of Technology, Eindhoven, the Netherlands
[3]Academic Center for Epileptology Kempenhaeghe & Maastricht UMC+, Heeze, the Netherlands
[4]Department of Biomedical Engineering, Eindhoven University of Technology, Eindhoven, the Netherlands

*** Correspondence:** Shouliang Qi, Sino-Dutch Biomedical and Information Engineering School, Northeastern University, Zhihui Street 500, Shenyang, 110167, China
qisl@bmie.neu.edu.cn

1. Supplementary Figures

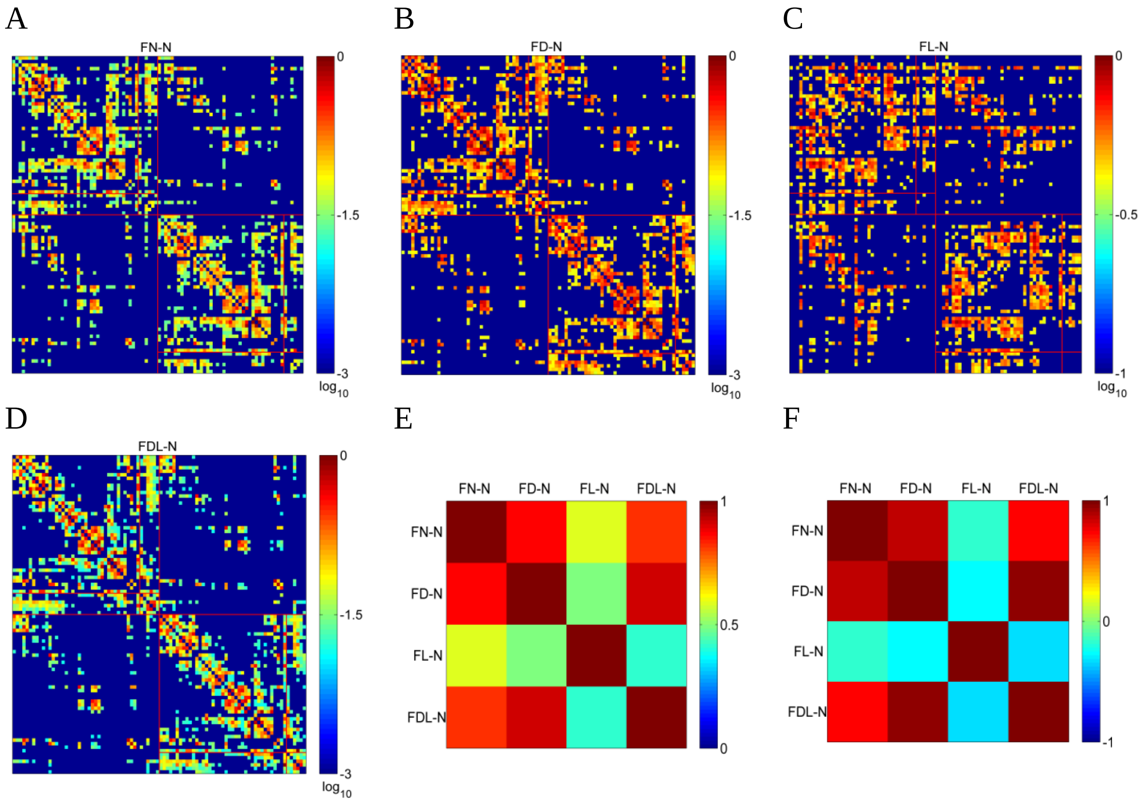

Supplementary Figure 1. Adjacency matrices of the non-optimized networks with various weighting methods and their relationships. (A) FN-N, the fiber number weighted network. (B) FD-N, the fiber density weighted network. (C) FL-N, the fiber length weighted network. (D) FDL-N, the network weighted by the fiber density corrected by the fiber length. (E) The overlapping ratio of edges of two weighting methods. (F) The correlation between each pair of weighting methods.

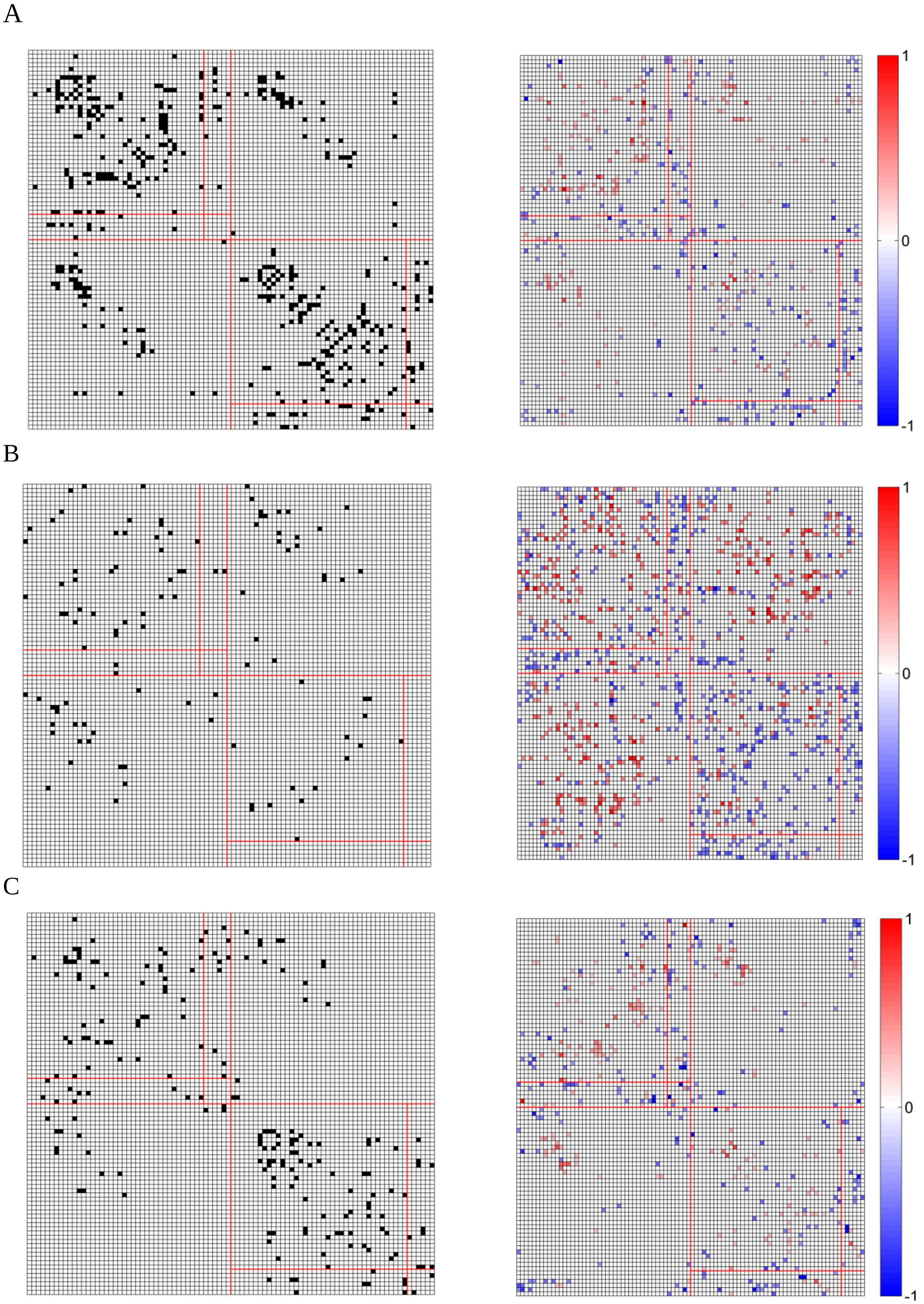

Supplementary Figure 2. Differences between the edges of the optimized and non-optimized networks at the sparsity of 0.75. (A) FD-N; (B) FL-N. (C) FDL-N. Right column shows the edges whose weights are significantly different in the optimized and non-optimized networks (The edge with no significant difference (*p*>0.05) is shown in white, and vice verse.). Here the non-zero edges in the optimized network are selected as the comparison reference. The right column show the situation of the false positive edges (in red) and the possible missing edges (in blue).

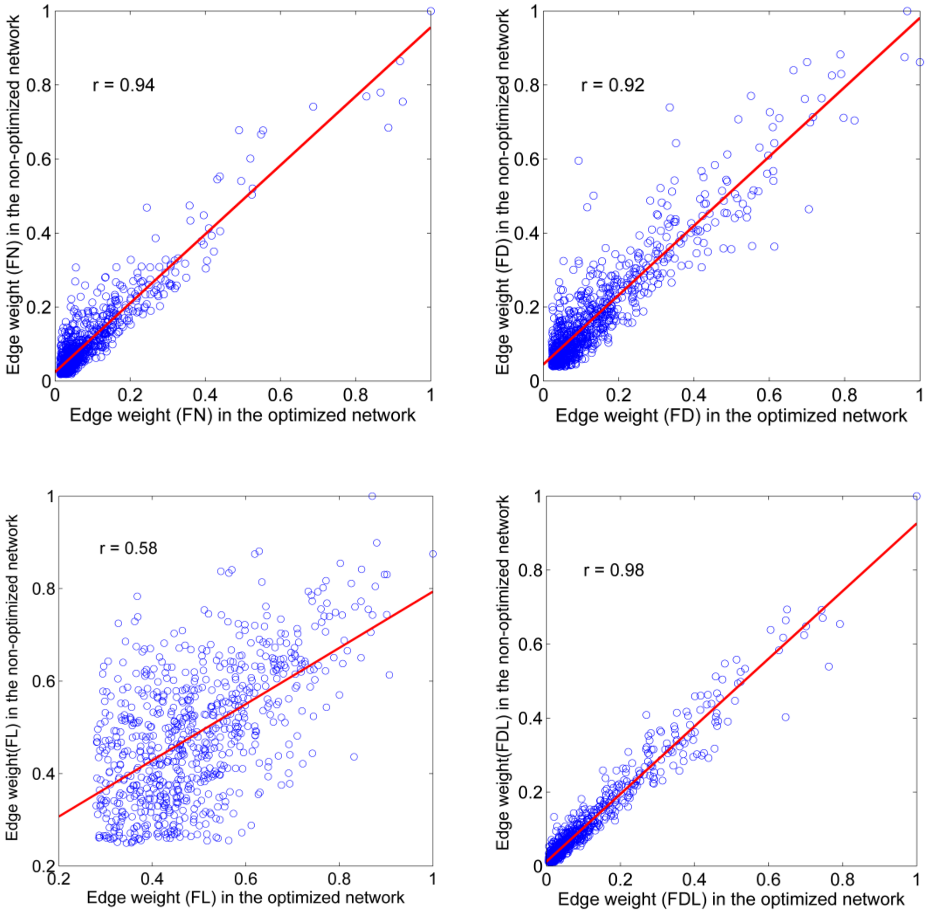

Supplementary Figure 3. Correlations between the edge weights of the non-optimized and optimized networks.

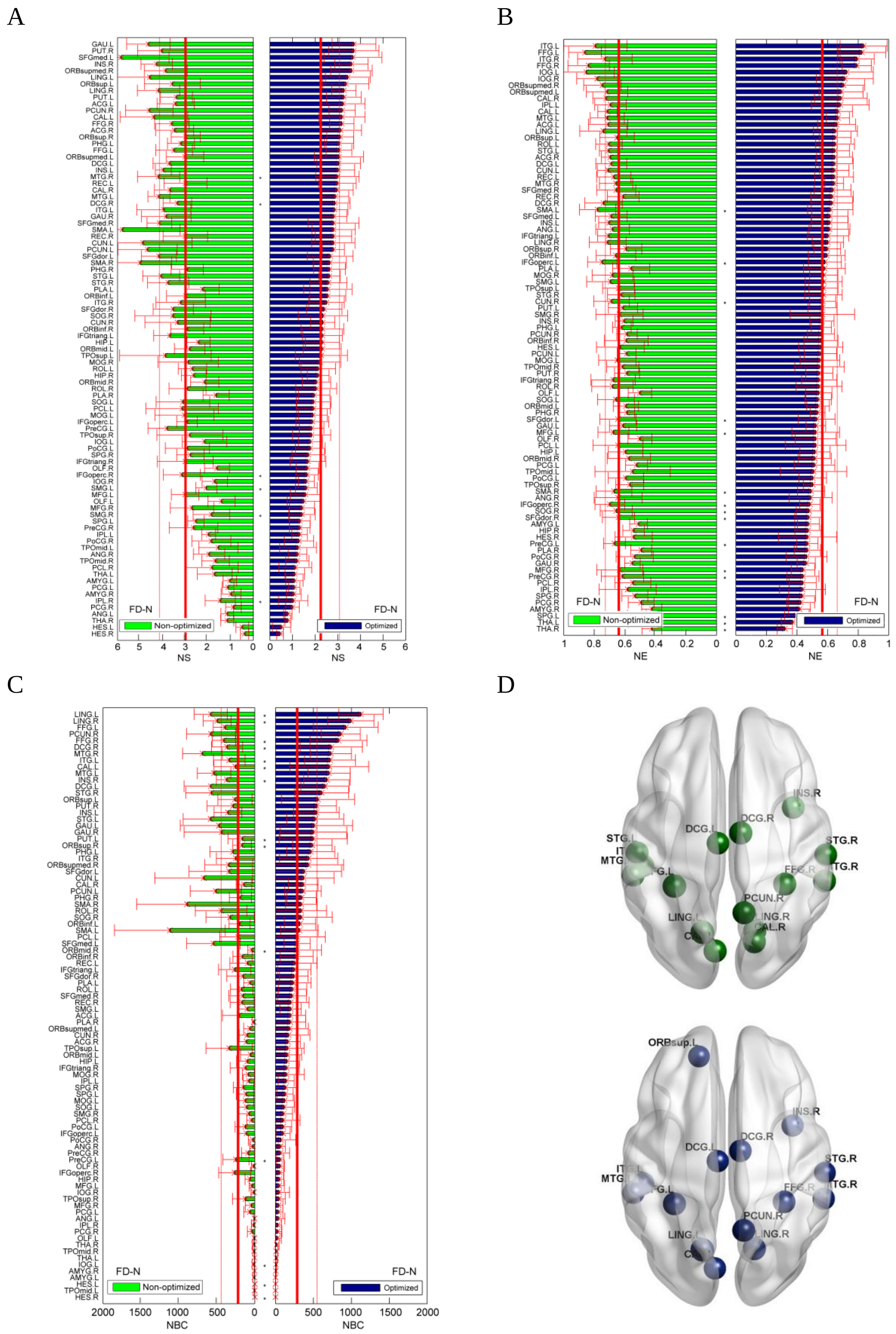

Supplementary Figure 4. Differences of the nodal measures between the optimized and non-optimized networks weighted by the fiber density (FD-N). Here * indicates there is a significantly difference between the measures from the non-optimized network and the optimized network ($p<0.05$). The vertical thin and bold lines indicate the $mean$ and $mean + SD$ of the measures of all nodes. (A) The node strength (NS). (B) The node efficiency (NE). (C) The node betweenness centrality (NBC). (D) The nodes with high NBC ($> mean + SD$) (The first row is for the non-optimized network and the second row is for the optimized network).

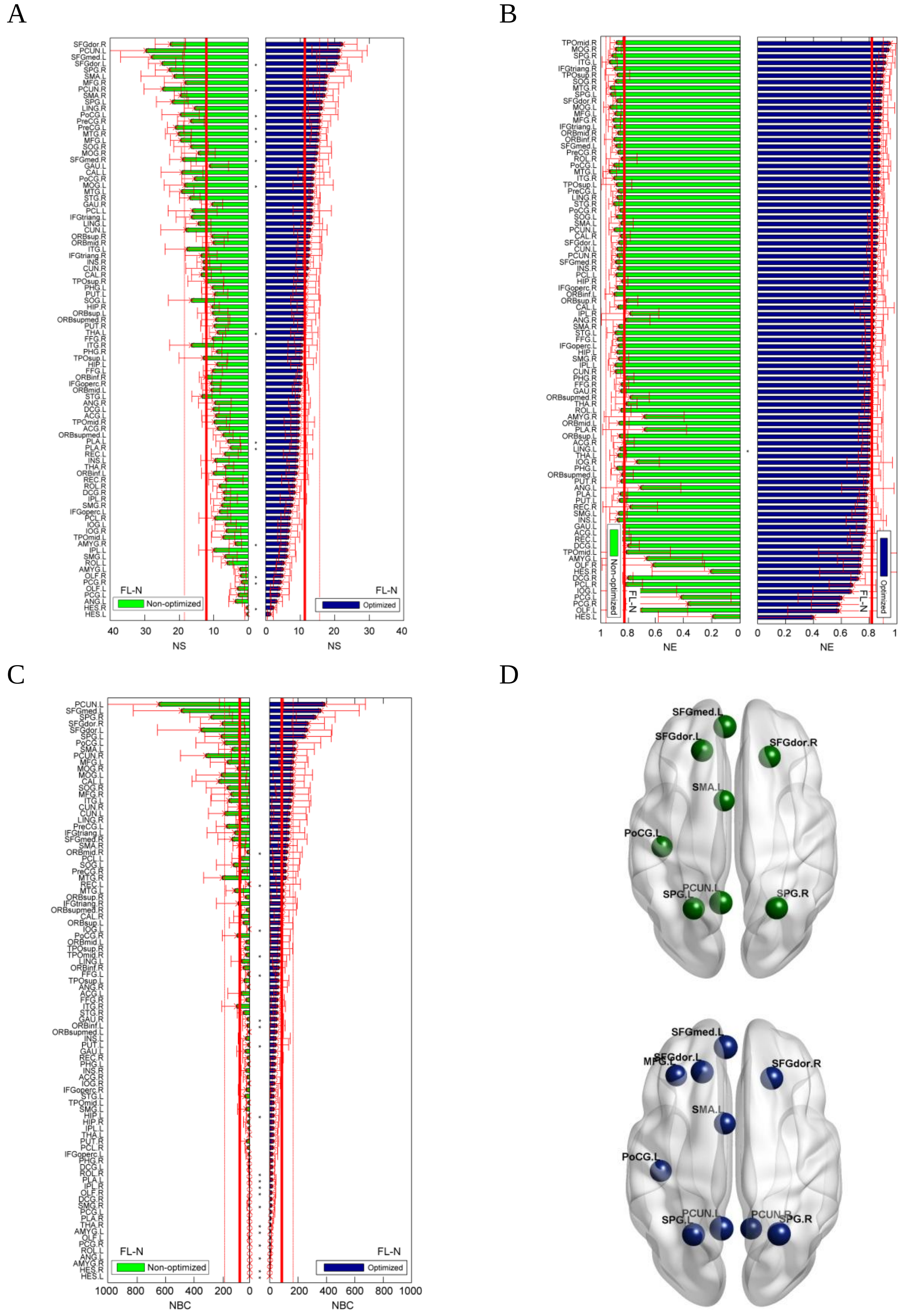

Supplementary Figure 5. Different of the nodal measures between the optimized and non-optimized networks weighted by the fiber length (FL-N). Here * indicates there is a significantly difference between the measures from the non-optimized network and the optimized network ($p<0.05$). The vertical thin and bold lines indicate the $mean$ and $mean + SD$ of the measures of all nodes. (A) The node strength (NS). (B) The node efficiency (NE). (C) The node betweenness centrality (NBC). (D) The nodes with high NBC ($> mean + SD$) (The first row is for the non-optimized network and the second row is for the optimized network).

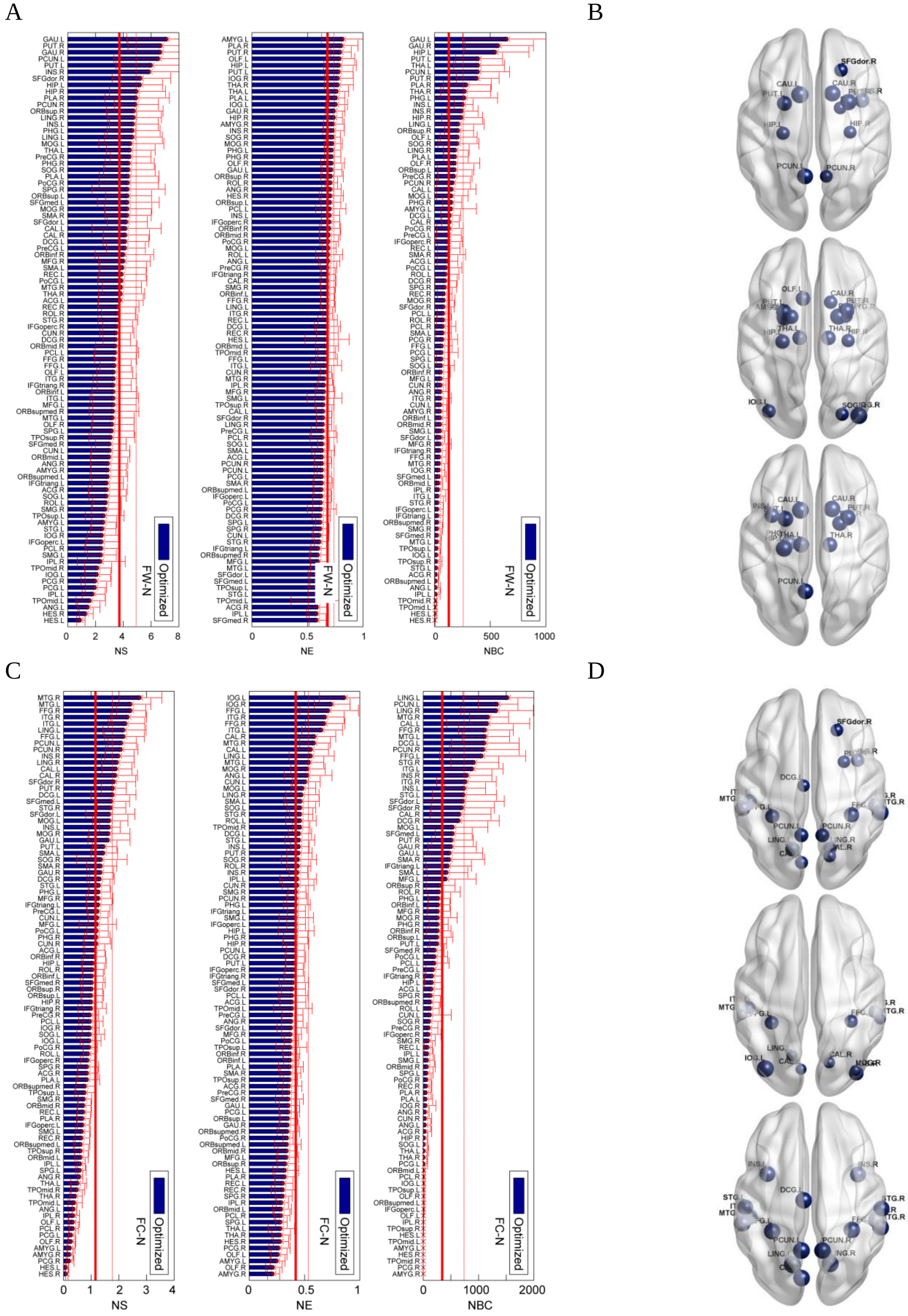

Supplementary Figure 6. The nodal measures of the optimized networks. (A) The optimized network weighted by the fiber weight (FW-N). (B) The nodes with high NS (the first row), NE (the second row) and NBC (the third row) ($> mean + SD$) in FW-N. (C) The optimized network weighted by the fiber contribution (FC-N). (D) The nodes with high NS (the first row), NE (the second row) and NBC (the third row) ($> mean + SD$) in FC-N.

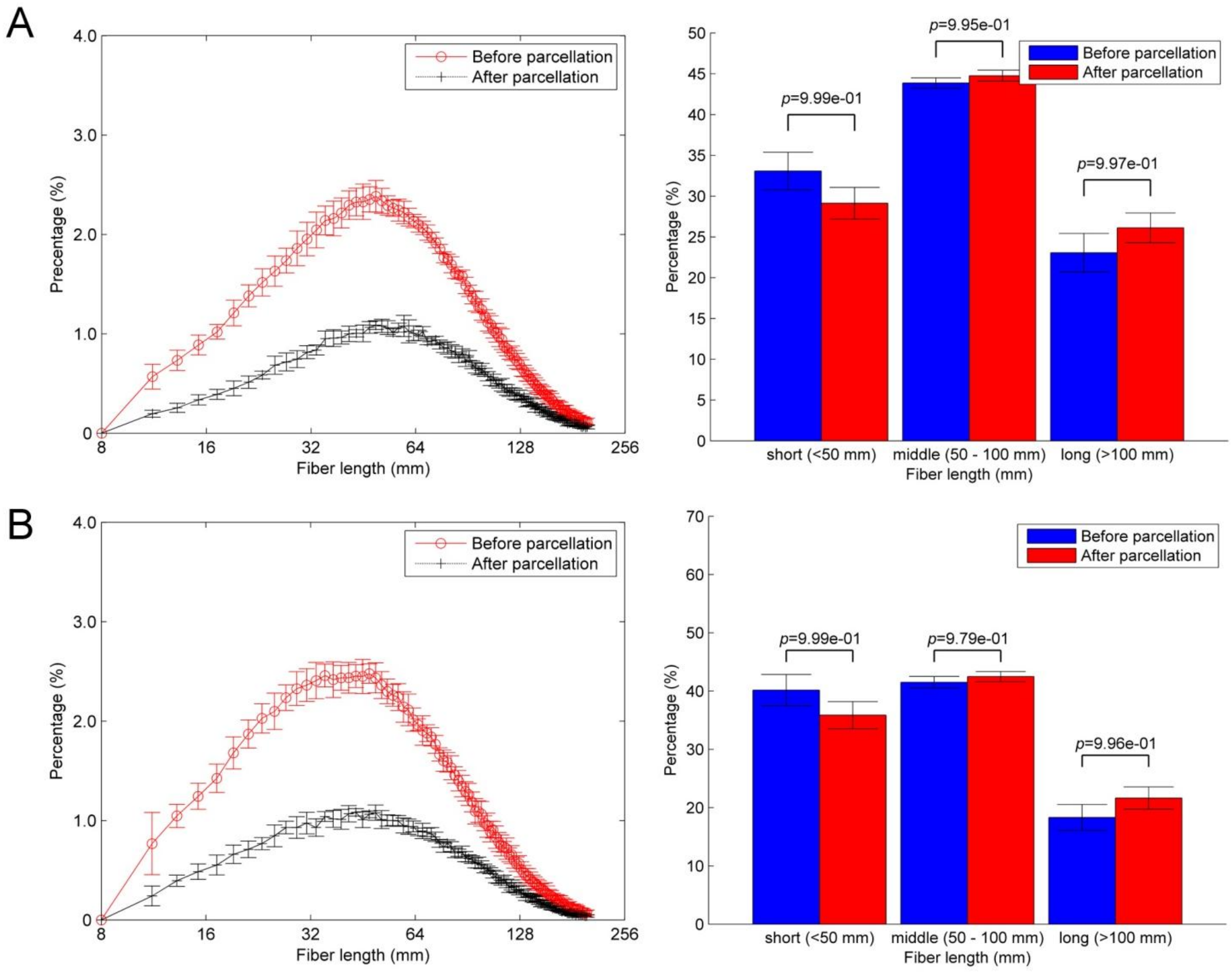

Supplementary Figure 7. Fiber length distributions for HCP dataset. (A) The fiber length distribution of the non-optimized connectome before and after parcellation. Left column is the histogram where the mean and standard deviation are plotted for 9 healthy subjects. Right column indicates the percentages of short, middle and long fibers. (B) The fiber length distribution of the optimized connectome before and after parcellation.

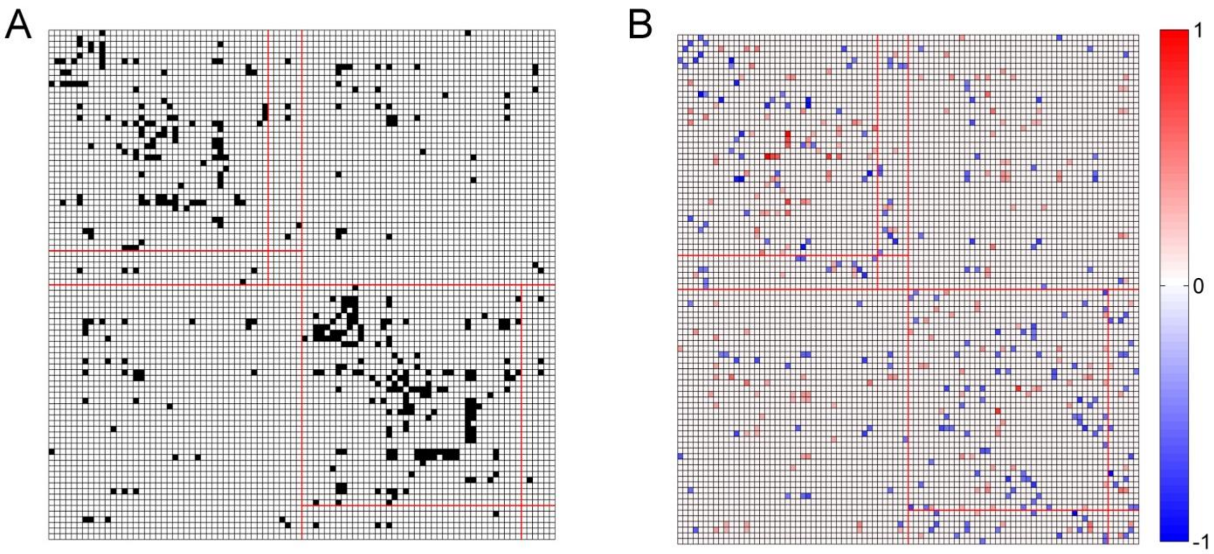

Supplementary Figure 8. Differences between the edges of the optimized and non-optimized networks weighted by the fiber number (FN-N) for HCP dataset. (A) The difference between the weighted edges in the optimized and non-optimized networks at a sparsity of 0.75 (The edges with significant difference ($p<0.01$) are shown in black.). (B) False positive edges and possible missing edges at a sparsity of 0.75. The legend at right shows the normalized weights, and the colors of red and blue indicate the false positive edges and possible missing edges, respectively.

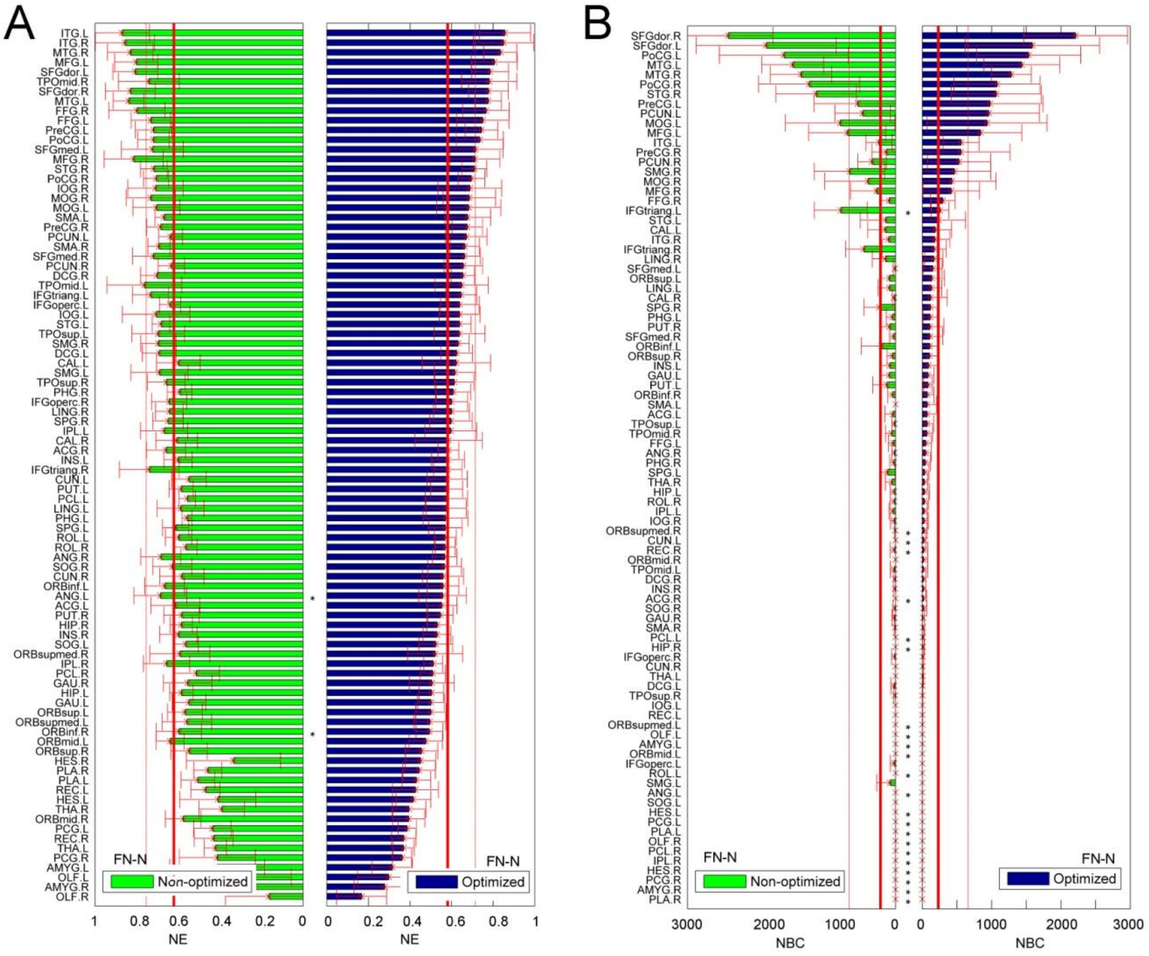

Supplementary Figure 9. Differences of the nodal measures between the optimized and non-optimized networks weighted by the fiber number (FN-N) for HCP dataset. Here* indicates there is a significantly difference between the measures from the non-optimized network and the optimized network ($p<0.05$). The nodes are ordered according to the nodal measures of the optimized network. The vertical thin and bold lines indicate the $mean$ and $mean + SD$ of the measures of all nodes. (A) The node efficiency (NE). (B) The node betweenness centrality (NBC)..
